# Crystal Structure of a Four-Layer Aggregate of Engineered TMV CP Implies the Importance of Terminal Residues for Oligomer Assembly

**DOI:** 10.1371/journal.pone.0077717

**Published:** 2013-11-04

**Authors:** Xiangyang Li, Baoan Song, Xi Chen, Zhenchao Wang, Mengjiao Zeng, Dandan Yu, Deyu Hu, Zhuo Chen, Linhong Jin, Song Yang, Caiguang Yang, Baoen Chen

**Affiliations:** 1 State Key Laboratory Breeding Base of Green Pesticide and Agricultural Bioengineering, Key Laboratory of Green Pesticide and Agricultural Bioengineering, Ministry of Education, Guizhou University, Guiyang, China; 2 School of Chemistry and Chemical Engineering, South China University of Technology, Guangzhou, China; 3 Shanghai Institute of Materia Medica, Chinese Academy of Sciences, Shanghai, China; University of Westminster, United Kingdom

## Abstract

**Background:**

Crystal structures of the tobacco mosaic virus (TMV) coat protein (CP) in its helical and disk conformations have previously been determined at the atomic level. For the helical structure, interactions of proteins and nucleic acids in the main chains were clearly observed; however, the conformation of residues at the C-terminus was flexible and disordered. For the four-layer aggregate disk structure, interactions of the main chain residues could only be observed through water–mediated hydrogen bonding with protein residues. In this study, the effects of the C-terminal peptides on the interactions of TMV CP were investigated by crystal structure determination.

**Methodology/Principal Findings:**

The crystal structure of a genetically engineered TMV CP was resolved at 3.06 Å. For the genetically engineered TMV CP, a six-histidine (His) tag was introduced at the N-terminus, and the C-terminal residues 155 to 158 were truncated (N-His-TMV CP^19^). Overall, N-His-TMV CP^19^ protein self-assembled into the four-layer aggregate form. The conformations of residues Gln36, Thr59, Asp115 and Arg134 were carefully analyzed in the high radius and low radius regions of N-His-TMV CP^19^, which were found to be significantly different from those observed previously for the helical and four-layer aggregate forms. In addition, the aggregation of the N-His-TMV CP^19^ layers was found to primarily be mediated through direct hydrogen-bonding. Notably, this engineered protein also can package RNA effectively and assemble into an infectious virus particle.

**Conclusion:**

The terminal sequence of amino acids influences the conformation and interactions of the four-layer aggregate. Direct protein–protein interactions are observed in the major overlap region when residues Gly155 to Thr158 at the C-terminus are truncated. This engineered TMV CP is reassembled by direct protein–protein interaction and maintains the normal function of the four-layer aggregate of TMV CP in the presence of RNA.

## Introduction

The tobacco mosaic virus (TMV) coat protein (CP) assembly system is composed of inlaid hydrophobic and hydrophilic patches with TMV RNA associated via multiple aromatic interactions between neighboring subunits to stabilize a helical virus structure [1]–[5]. Several experiments to crystallize TMV CP have demonstrated a dimer of bilayer disks with 17 subunits per layer, and the structures have been resolved to show the total form of 34 subunits [6]–[10]. Each subunit can be split into three regions: the high radius (HR), the middle radius (MR), and the low radius (LR) regions [11]. The HR region is far from the central axis of TMV CP, whereas the MR and LR regions are close to the central axis. The first structural determination of TMV CP, solved by X-ray fiber diffraction at a resolution of 2.8 Å in 1978 [11], reveals a similar conformation of protein subunits in the two layers. In 1998, a four-layer aggregate of TMV CP without RNA was reported [12], revealing an important role of water in its biological macromolecular assembly. The TMV viral structure was determined in 1989 through X-ray fiber diffraction [13], suggesting that viral disassembly is driven by electrostatic repulsions between nucleic acids and proteins. However, the electron densities of the C-terminal residues 155 to 158 are poor and display a high B factor. In 2007, the molecular assembly of TMV CP and RNA was described using cryo-electron microscopy (cryo-EM) at 4.6 Å. Notably, this assembly was more ordered: the residues clearly showed secondary structure near the RNA molecule in the LR region [14]. In this complex, the electrostatic interactions between the phosphate backbone of the RNA and the arginine residues (Arg112 and Arg113) significantly contributed to the stability of the complex. Taken together, the main differences between the previously determined structures of the virus and TMV CP protein lie in the LR region. In the virus particle, the interactions between the RNA and Arg90 and Arg92 could be observed by the cryo-EM method, but not by the X-ray crystallographic method. The interactions were more ordered in the LR region of the virus. In addition, the contact pair of Glu50 and Asp77 in the MR region was found to play an important role in the assembly and disassembly of the virus [15]–[19].

Here, we identify the effect of the C-terminal peptides on the molecular packaging and interactions of subunits within TMV CP aggregates. In order to purify and crystallize TMV CP, we constructed a TMV CP variant with a His-tag at the N-terminus and a truncation of residues Gly155 to Thr158 at the C-terminus. The engineered N-His-TMV CP^19^ protein was crystallized using hanging-drop vapor diffusion and the micro-seeding method. The crystal structure of the four-layer aggregate of N-His-TMV CP^19^ was determined at 3.06 Å. The major differences between N-His-TMV CP^19^ and the previously reported TMV CP structures are the manner of subunit assembly and the protein-protein interactions in the major overlap region. It should be noted that this engineered truncation mutant still maintains the normal function of the four-layer aggregate of TMV CP in the presence of RNA.

## Results and Discussion

### The Oligomeric State of N-His-TMV CP^19^ in Solution

To prepare N-His-TMV CP^19^ for crystallization, four amino acids (Gly155, Pro156, Ala157 and Thr158) were truncated at the C-terminus, and a 6-His-tag was incorporated at the N-terminus. Freshly purified protein was examined in solution using size exclusion chromatography (SEC). In 20 mM sodium phosphate and 100 mM NaCl (pH 8.0) (phosphate buffer; PB), the retention time of the N-His-TMV CP^19^ protein revealed its oligomeric state as a dimer and monomer. After equilibration in a buffer containing 300 mM ammonium sulfate and 100 mM Tris-HCl (pH 8.0), the oligomeric state of N-His-TMV CP^19^ was converted to a 20S structure. These results show that the assembly of N-His-TMV CP^19^ is influenced by the environmental conditions.

### Crystallization of N-His-TMV CP^19^ Using a Micro-Seeding Method

Several TMV CPs have previously been crystallized by the micro-dialysis method against a buffer containing 300 mM ammonium sulfate and 100 mM Tris-HCl (pH 8.0) [20]–[22]. However, crystallization using this method is proven to be erratic in terms of reproducibility and crystal morphology. Instead, we crystallized N-His-TMV CP^19^ using hanging-drop vapor diffusion and a micro-seeding method. The micro-seeding method facilitated efficient and reproducible crystal growth of N-His-TMV CP^19^ [23]. This crystallization procedure was successfully used to produce large crystals of N-His-TMV CP^19^ with a typical maximal dimension of 0.2 mm ×0.3 mm within several days [24].

### Overall Structure of N-His-TMV CP^19^

**Figure 1:**
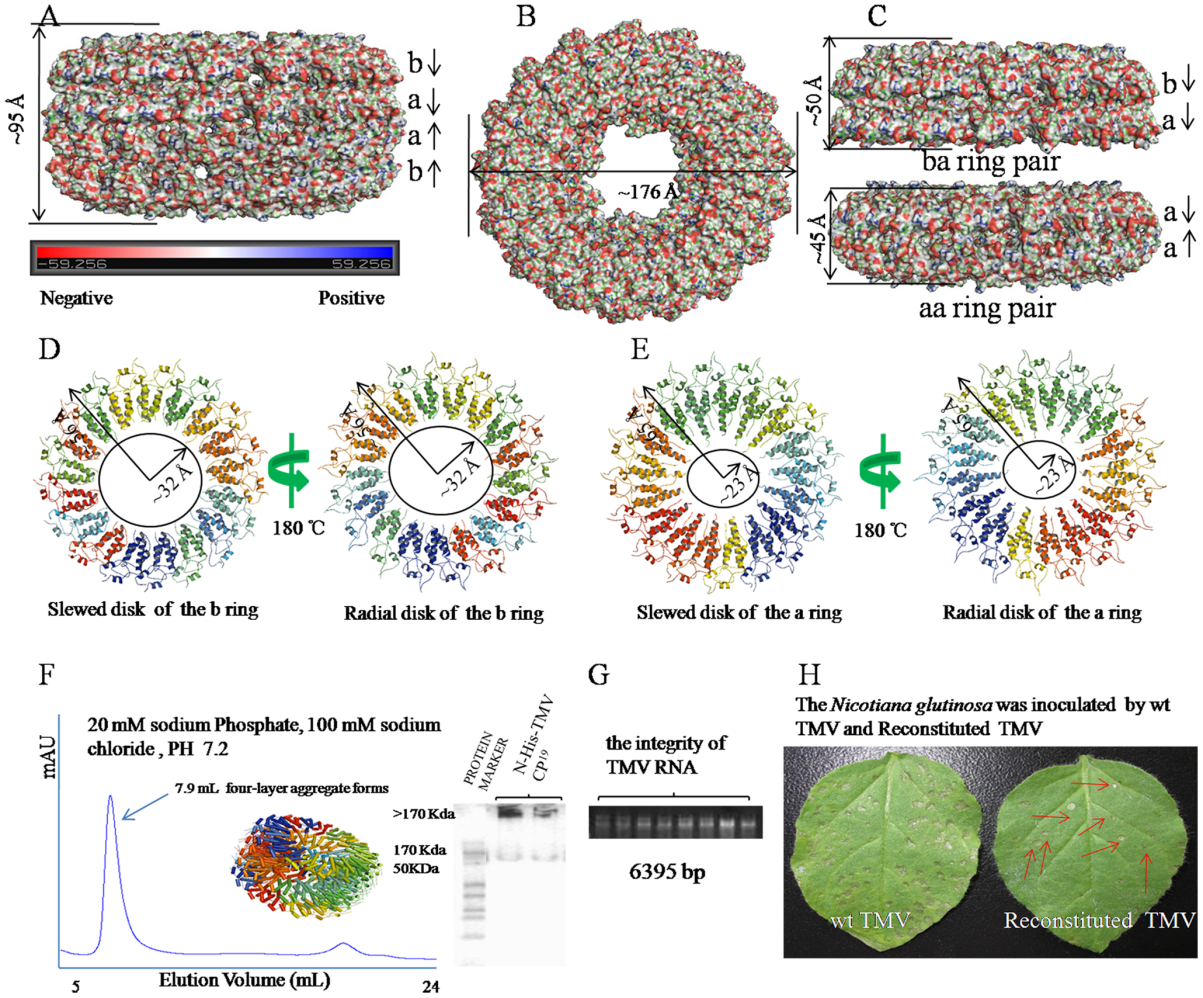
Crystal structure and function of the four-layer aggregate disk of N-His-TMV CP^19^. (**A**) Electrostatic surface presentation of the N-His-TMV CP^19^ four-layer aggregate of ∼95 Å in height. The electrostatic surface was calculated in pyMOL. The complete four-layer aggregate is shown in four rings in the following order: b-ring, a-ring, a-ring, b-ring. (**B**) Electrostatic surface presentation of the N-His-TMV CP^19^ four-layer aggregate disk of ∼176 Å in diameter. (**C**) Electrostatic surface presentation of the ba-ring and aa-ring pairs. The aa-ring pair is sandwiched between the two b-rings. The a-ring and a-ring chains are anti-parallel and pack more tightly to ∼45 Å in height; the b-ring and a-ring chains are parallel and pack to ∼50 Å in height. (**D**) The slewed (radial) disk of the b-ring defines a 64 Å diameter axial pore based on residue Val 114 (bottom panel); (**E**) the slewed (radial) disk of the a-ring defines a 46 Å diameter axial pore based on residue Thr107 (bottom panel). (**F**) Assembly of the N-His-TMV CP^19^ four-layer aggregate, as measured by SEC and native-PAGE electrophoresis. (**G**) TMV RNA integrity was examined by 1% agarose gel electrophoresis. (**H**) The *N. glutinosa* was inoculated with WT TMV and reconstituted TMV. The local lesions that were inoculated with reconstituted TMV are labeled with red arrows.

**Figure 2:**
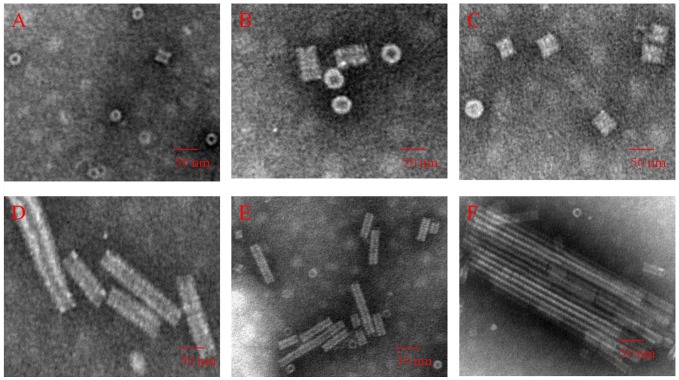
Characterization of the disk and helical stack height using TEM in 10 mM PB, 100 mM NaCl, pH 7.2 with increasing concentrations of protein, temperature and time. As concentration, temperature and time increased, the dominant structure changed from disks to rods. (A) 1.8 mg/mL N-His-TMV CP^19^, 4°C, 10 h: monomers, dimers and disks. (B) 1.8 mg/mL N-His-TMV CP^19^, 20°C, 15 h: disks. (C) 1.8 mg/mL N-His-TMV CP^19^, 20°C, 20 h: disks. (D) 14 mg/mL N-His-TMV CP^19^, 20°C, 10 h: disks and rods. (E) 14 mg/mL N-His-TMV CP^19^, 20°C, 15 h: disks and rods. (F) 14 mg/mL N-His-TMV CP^19^, 20°C, 20 h: rods.

**Figure 3:**
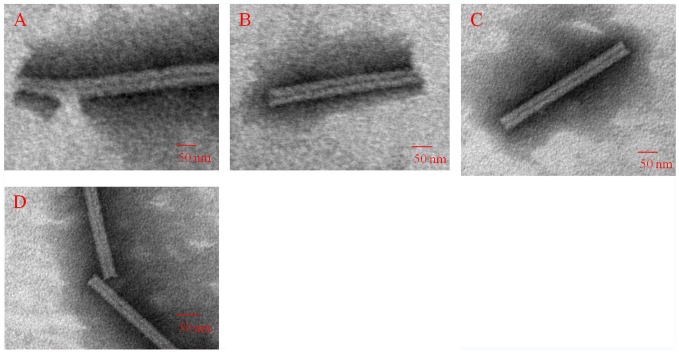
Characterization of reconstituted N-His-TMV CP^19^ virus using TEM in 10 mM PB, 100 mM NaCl, pH 7.2 after 20 h at 22°C. 1 mL purified self-assembled N-His-TMV CP^19^ disks (1.8 mg/mL) in 10 mM PB, 100 mM NaCl, pH 7.2 was mixed with 0.2 mL purified TMV RNA for 20 h at 22°C. Four TEM images of the reconstituted virus are shown in A–D.

The N-His-TMV CP^19^ crystals belong to an orthorhombic space group P2_1_2_1_2, with unit cell dimensions a = 173 Å, b = 222 Å, and c = 226 Å (**Table S1 and Figure S1**). The structure was solved by molecular replacement and refined to a resolution of 3.06 Å (PDB code 4GQH). The local noncrystallographic symmetry (NCS) axis was refined with a grid search wherein we varied the orientation or translation of the asymmetrical unit using XPLOR rigid body dynamics [12]. By using the NCS restraints option within the XPLOR refinement program, the 34 subunits of the asymmetrical unit were individually refined by simulated annealing. There were no departures from the NCS found during this procedure of refinement, as indicated by the Root Mean Square Deviation (RMSD) between the individual subunits (0.01–0.02 Å), The secondary structures of the a-chain, the b-chain, and the b- and a-chain in our refined atomic model are very similar to ones described by Bhyravbhatla et al. [12], which have RMSDs of ∼0.6 Å, ∼1.6 Å and ∼1.6 Å for the carbon-alpha backbone (not including the loop region and carboxyl-terminal four residues) (**Figure S2A**, **Figure S2B** and **Figure S2C**). The well-ordered portions of the virus subunit [19] have similar secondary structure when compared with either of the a-chains in the disk aggregate; superposition of the core of the protein backbone carbon-alpha atoms (RMSD of ∼1.1 Å) establishes that the overall folding of the protein is similar in the TMV CP helical structure and the TMV CP disk structure (**Figure S2D**). The ordered segments of the a- and b-chains can be superimposed on each other by a rigid body translation with a RMSD of 0.2 Å for the carbon-alpha atoms (**Figure S3**). The present atomic model also details the locations and orientations of the side chains involved in the inter- and intra-subunit interactions based on the error estimated by Luzzati statistics [25]. The refinement statistics after adding NCS restraints or constraints are listed in **Table S1**. The HR and LR residue densities corresponding to fitted coordinates are supplied in **Figure S4 and Figure S5**. It is clear that the density for the large hydrophobic residues, some of the small hydrophobic residues and the basic side chains are in agreement with the fitted coordinates. The N-His-TMV CP^19^ can form disks and infectious viruses (**Figure S6 and Figure S7**). The density corresponding to the side chains of N-His-TMV CP^19^ is mapped in **Figure S8 and Figure S9**. The 2Fo-Fc electron density maps have been contoured at 1.0 σ level, and the residues have been shown according to structure sequence. Similar to previous studies, one asymmetric unit was observed containing four aggregate disks, with every disk containing 17-fold monomers [11], [12]. The structure of the N-His-TMV CP^19^, which contains a hexahistidine tag at the N-terminus to facilitate purification [26], is similar to the four-layer aggregate [12]. The N-His-TMV CP^19^ four-layer aggregate was about ∼176 Å in diameter (**Figure 1A**) and ∼95 Å in height (**Figure 1B**). The four-layer aggregate disk was packed into a ba-ring pair outside (∼50 Å in height) and an aa-ring pair in the centre (∼45 Å in height) (**Figure 1C**). In the HR region, a well-ordered electron density map corresponding to the protein was observed (**Figure S8A,** **Figure S4** and **Figure S5**). In the LR region, a poor-quality electron density corresponding to the protein was mapped (**Figure S8B, Figure S4 and Figure S5**). Therefore, residues 91–113 are absent in the b-ring atomic model, and residues 92–106 are absent in the a-ring atomic model (**Figure 1D** and **Figure 1E**). The slewed (radial) disk of the b-ring defines an axial pore of 64 Å in diameter based on residue Val114 (bottom panel); and the slewed (radial) disk of the a-ring defines an axial pore of 46 Å in diameter based on residue Thr107 (bottom panel). Residues Gly155, Pro156, Ala157 and Thr158 at the C-terminus of the N-His-TMV CP^19^ chains are truncated, which may facilitate the formation of the four-layer aggregate detected by SEC (20 mM PB, 100 mM NaCl, PH 7.2), and native-PAGE and TEM, after equilibration with an ammonium sulfate solution as above (**Figure 1F** and **Figure S7**). N-His-TMV CP^19^ refolding and further self-assembly was carried out at a protein concentration of 1.8 mg/mL. When the N-His-TMV CP^19^ was incubated at a concentration of 1.8 mg/mL at 22°C for 5 hr, the N-His-TMV CP^19^ disk or helical structures were formed as observed using TEM in solution (**Figure 2**). When TMV RNA (**Figure 1G**) isolated from wild type (WT) TMV was added to the N-His-TMV CP^19^ disk or helical structure, a reconstituted TMV particle was formed (**Figure 3**). This virus particle can infect *Nicotiana glutinosa* (**Figure 1H** **and Figure S6**). Thus, the N-His-TMV CP^19^ four-layer aggregate can effectively package and protect the nucleic acid under conditions of 20 mM PB, 100 mM NaCl and pH 7.2. For infection of tobacco leaves, the TMV CP disks undergo disassembly and reassembly mediated by RNA, and the reconstituted virus-like particles are made up of the helical form as determined by TEM (**Figure 3** **and Figure S6**). In other words, the RNA can stabilize the disordered residues in the LR region.

**Figure 4:**
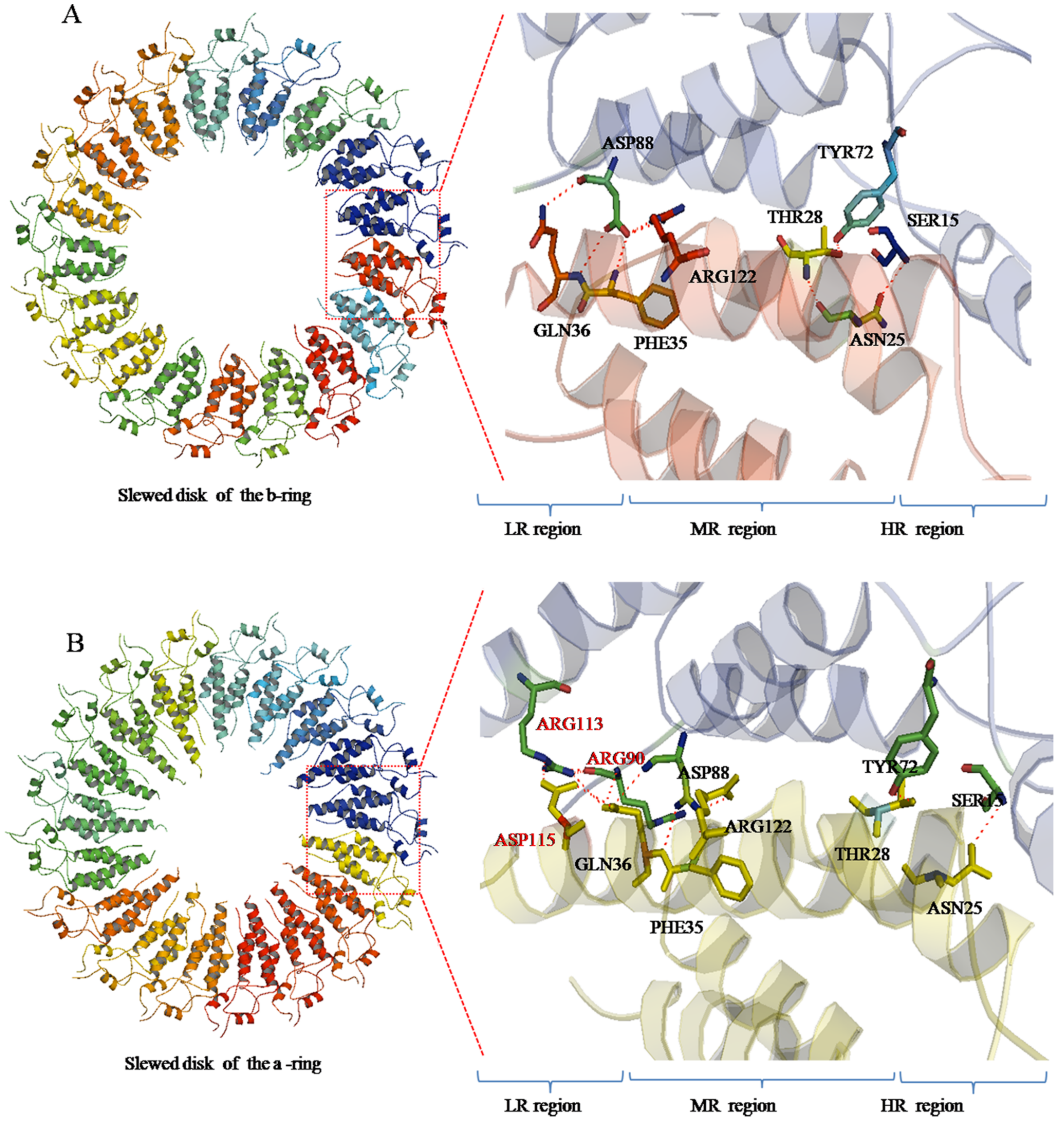
N-His-TMV CP^19^ interactions involved in b-ring and a-ring inter-subunits. (**A**) Inter-chain interactions between Asn25-Ser15, Tyr72-Thr28, Phe35-Asp88, Gln36-Asp88 and Arg122-Asp88 in slewed disks of the b-ring were observed. These interactions are different from the previously reported disk inter-chain interactions (mediated by water). (**B**) Because the aa-ring is sandwiched between the b-rings, the a-ring inter-subunits in the LR region loop residues have stronger interactions than the b-ring inter-subunits, except for the observed inter-chain interactions between Asn25-Ser15, Tyr72-Thr28, Phe35-Asp88, Gln36-Asp88 and Arg122-Asp88.

**Figure 5:**
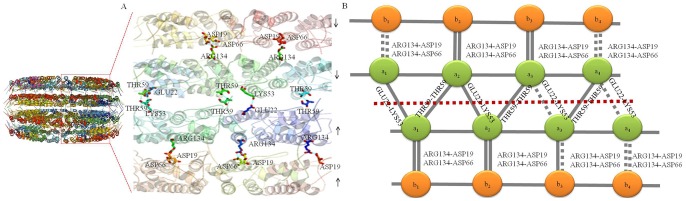
Side view of the major overlap region between layers of atomic chains. (**A**) The Asp19-Arg134 and Asp66-Arg134 protein–protein interactions between the b-pair and a-pair of atomic chains; the Lys53-Glu22 and Thr59-Thr59 protein–protein interactions between the a-pair and a-pair of atomic chains. (**B**) The N-His-TMV CP^19^ four-layer aggregate disk interaction model involves inter-subunits between layers. The horizontal dotted line is the central axis of the aa-pair. A particular slide range is observed because of the Thr59-Thr59 interaction.

In the atomic model, the b-ring and a-ring in the HR and MR region are packed in order with observable inter-chain interactions between Asn25-Ser15, Tyr72-Thr28, Phe35-Asp88, Gln36-Asp88 and Arg122-Asp88 (**Figure 4A**), and the residue densities corresponding to fitted coordinates are supplied in **Figure S10**, The inter-molecular hydrogen bonds between residues are indicated by magenta dashed lines, The residues are shown according to N-His-TMV CP^19^ sequence. The a-ring subunits in the LR region loop residues are of higher order than the b-ring subunits with observable inter-chain interactions between Asn25-Ser15, Tyr72-Thr28, Phe35-Asp88, Gln36-Asp88 and Arg122-Asp88 (**Figure 4B**). In the previous study by Bhyravbhatla et al [12], no observable inter-chain interactions were observed in the LR region. In the helical structure, the Arg113-Arg90 interaction was observed and these residue loops were stabilized by RNA base-binding pockets [13], [19]. There are obvious differences in the four-layer assembly in the current structure and the previously reported one. For N-His-TMV CP^19^, a six-histidine (His) tag was introduced at the N-terminus, and the C-terminal residues 155 to 158 were truncated; direct inter-chain interactions between Asn25-Ser15, Tyr72-Thr28, Phe35-Asp88, Gln36-Asp88 and Arg122-Asp88 were observed in N-His-TMV CP^19^ (**Figure S10**). Because the oligomer terminal residues were changed, the conformations of residues Gln36, Thr59, Asp115 and Arg134 in the HR and LR regions of N-His-TMV CP^19^ were found to be significantly different from those observed in the helical structure (PDB code: 1EI7) (**Figure S11**) and in the four-layer aggregate disk structure of TMV CP (PDB code: 3JO6) (**Figure S12**). Protein–protein interactions on the salt bridges between Asp19 and Arg134, and between Asp66 and Arg134 were observed in the dihedral-related ba-chain pair. In the dihedral related aa-chain pair, the protein–protein interactions on the salt bridges between Lys53 and Glu22 and the hydrogen-bonding interactions between Thr59 and Thr59 were observed in the middle between trans-layers. All interactions between the four-layer aggregate disk had similar bond lengths (∼3.5 Å) (**Figure 5A**). A particular slide range was observed because of the interaction force between the a_1_-chain subunit Thr59 and the a_2_-chain subunit Thr59 in the HR region (**Figure 5B**). In contrast to the earlier model (PDB code: 1EI7), the protein–protein interactions within the salt bridges were mediated in most part by solvent molecules [12]. The electron density maps and fitted coordinates of direct protein–protein interactions involving salt-bridges between Asp19 and Arg134 are depicted in **Figure S13A**. The electron density map and fitted coordinates corresponding to the direct protein–protein interactions between Asp19-Arg134 and Asp66-Arg134 for the b-pairs and a-pairs of N-His-TMV CP^19^ and the protein–protein interactions between Asp19-Arg134 and Asp66-Arg134 for the previously reported TMV CP disk structures (PDB code: 1EI7) are depicted in **Figure S13B**. For the previously reported TMV CP disk structure, protein–protein interactions between Asp19-Arg134 and Asp66-Arg134 for the b-pairs and a-pairs were mediated by water. The electron density maps of protein–protein interactions involving salt-bridges between Lys53 and Glu22 are depicted in **Figure S13C**. The electron density maps of hydrogen-bonding interactions between Thr59 and Thr59 were observed in the middle trans-layers (**Figure S13D**). As shown in **Figure S11** and **Figure S14,** a close-up electron density map and fitted coordinates show the inter-chain residues of N-His-TMV CP^19^ and the previously reported TMV CP (PDB code: 1EI7) subunits. Compared to the previously reported structures, these direct hydrogen-bonding interactions between the protein–protein trans-layers were not observed. In the previous structure (PDB code: 1EI7), the protein–protein interactions involved salt-bridges mediated by water. For the N-His-TMV CP^19^ structure, the conformations of Asp19, Arg134, Asp66, Lys53, Glu22 and Thr59 were different from the previously reported structure (**Figure S11** and **S14**).

**Figure 6:**
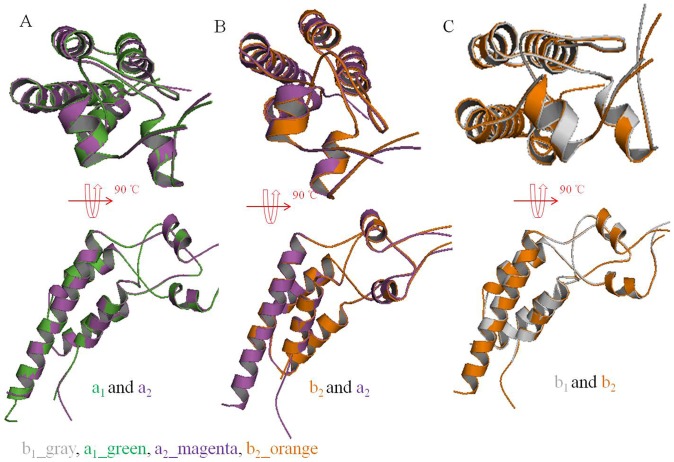
Superposition of two monomers of N-His-TMV CP^19^. (A) Superposition of monomers between the a_1_-chain and a_2_-chain in N-His-TMV CP^19^. (B) Superposition of monomers between the b_2_-chain and a_2_-chain in N-His-TMV CP^19^. (C) Superposition of monomers between the b_1_-chain and b_2_-chain in N-His-TMV CP^19^.

However, there were no differences in the N-His-TMV CP^19^ protein monomers. The N-His-TMV CP^19^ protein monomers could assemble into dimers, oligomers, a or b disks and four-layer aggregates. The LR region loops of the b disk were lost more than the a disk. Every two monomers were aligned between the aa disks, ab disks and bb disks. We found that there were no differences for the CP protein monomers (**Figure 6**).

### Comparison with the Reported TMV CP Structures

**Figure 7:**
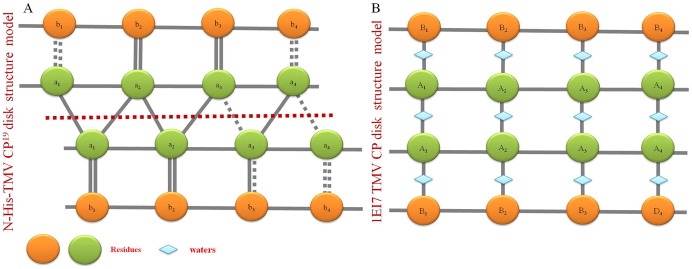
The inter-chain and intra-chain interaction model of N-His-TMV CP^19^ and the previously reported TMV CP disk (PDB code: 1EI7). (A) The inter-chain and intra-chain interaction model of N-His-TMV CP^19^. (B) The inter-chain and intra-chain interaction model of the previously reported TMV CP disk structure (PDB code: 1EI7).

The amino acid sequence of N-His-TMV CP^19^ is consistent with the previously reported sequence of the TMV CP disks and TMV CP helices except that a six-His tag was included at the N-terminus and four residues were truncated at the C-terminus, the atomic model of N-His-TMV CP^19^ presented here includes a newly formed section between the LR loop region and water molecules as observed by electron density mapping (**Figure S4**). Compared with the previously reported disk structure (PDB code: 1EI7) and the helical structure (PDB code: 3JO6), the major differences are the manner of subunit assembly and protein-protein interactions in the major overlap region. In the N-His-TMV CP^19^ structure, most protein–protein interactions were observed in the N-His-TMV CP^19^ inter-chains within 3.5 Å, including salt bridges between Asp19-Arg134 (∼3.12 Å), Asp66-Arg134 (∼3.29 Å), Lys53-Glu22 (∼3.38 Å), Arg122-Asp88 (∼2.43 Å), Arg113-Asp115 (∼3.24 Å) and hydrogen bonding of Asn25-Ser15 (∼2.73 Å), Tyr72-Thr28 (∼2.76 ), Phe35-Asp88 (∼3.03 Å), Gln36-Asp88 (∼3.50 Å) and Thr59-Thr59 (∼3.28 Å). In the previously reported disk structure (PDB code: 1EI7), the charged groups of the interactions were more than 4.0 Å (for example, the Asp66-Arg134 pair was ∼4.2 Å, and the Glu22-Lys53 pair was ∼5.4 Å), and most side-chains of the residues interacted via salt bridges and hydrogen-bonding through a network mediated by water [12], A model of these CP disks is provided in **Figure 7**. Furthermore, for our structure, the N-His-TMV CP^19^ b-ring residues 91–113 (a-ring residues 92–106) in the low-radius inner loop region were missing, which is dissimilar to the previously reported TMV CP disk and helical structures. In the TMV CP disk structure (PDB code: 1EI7), the a-ring and b-ring residues 90–110 in the low-radius inner loop region were uniformly disrupted [12]. In the helical structure (PDB code: 3JO6), the residues 85–103 in the low-radius inner loop region were stabilized by RNA binding, and the residues Asp116-Arg92 conformation was affected by calcium-driven switches [19].

The structures of the side chains differed: a figure of the electron density map is depicted with an average resolution of 3 Å (**Figure S4**). In the atomic model, the region between 0 and 30 Å in radius, corresponding to the central 90-Å-diameter hole in the macromolecular assembly, was completely filled with solvent. In the region between 30-Å and 40-Å in radius, the atomic chain was poorly ordered with no observable inter-chain or intra-chain interactions. In the initial study done by Bhyravbhatla et al. [12], no electron density corresponding to the protein was observed in this region. Therefore, the N-His-TMV CP^19^ b-ring residues 91–113 (a-ring residues 92–106) are absent in the atomic model. Collecting good low-resolution data and using 17-fold redundancy allowed us to map out the backbone of the polypeptide chain in this region. The side chains are well defined in the electron density map in **Figure S5**.

**Figure 8:**
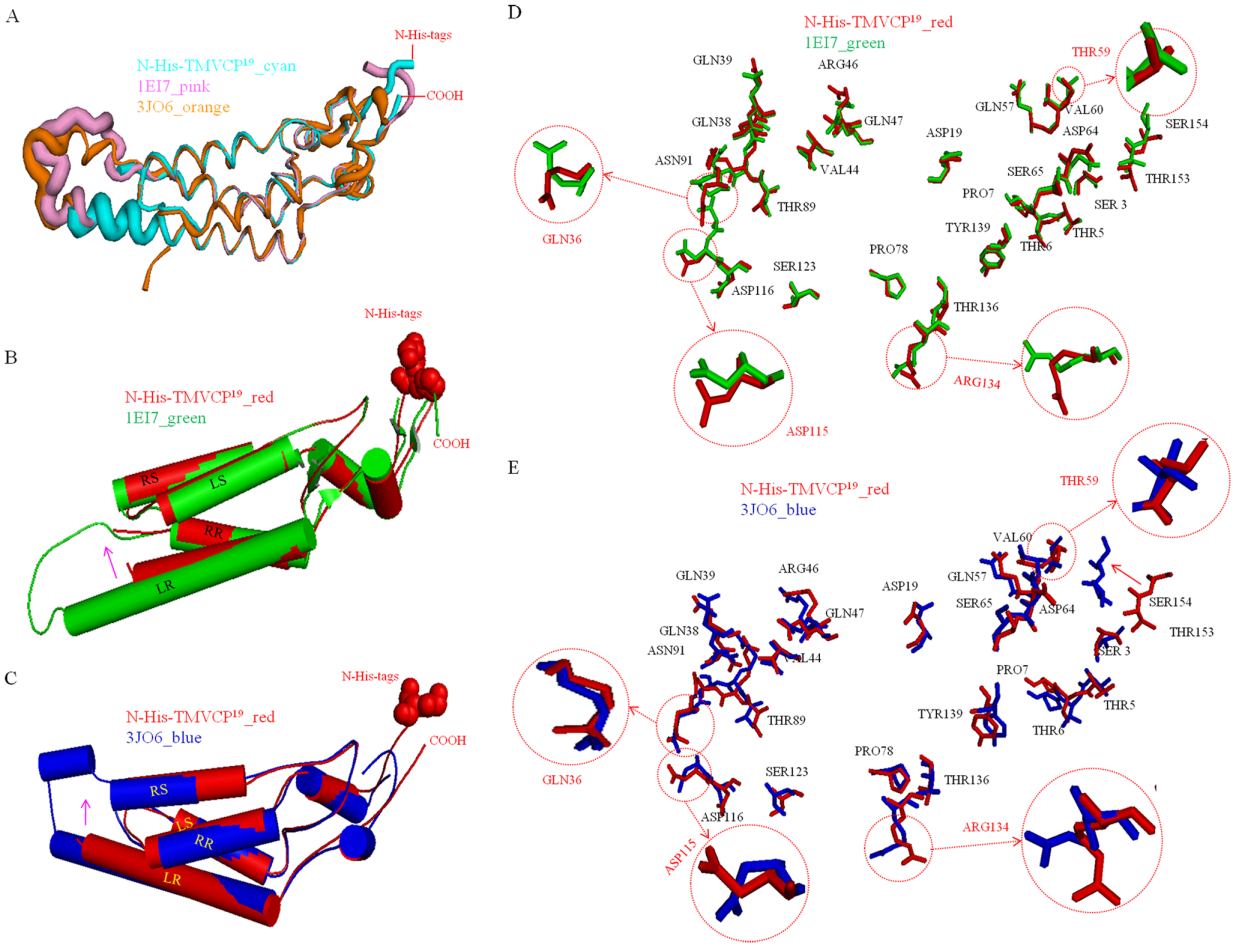
Structure comparisons between the previously reported TMV CP structure (PDB code 1EI7) and N-His-TMV CP^19^; and virus structure (PDB code 3JO6) and N-His-TMV CP^19^. (A) The B factors of the atomic model are based on the atomic structure. Compared with TMV CP (PDB code 1EI7) and TMV CP (PDB code 3JO6), the N-His-TMV CP^19^ residues at the C-terminus loop (residues 148–154) have a low atomic B factor; the N-His-TMV CP^19^ in the low-radius inner loop [residues 104–111 of the a-ring (residues 107–111 of the b-ring)] have a high atomic B factor, indicating that the residues in these loops are disordered. In this structure, the disordered residues are stabilized by protein–protein hydrogen bonding interactions in the adjacent subunit. (B) Structure comparison between the TMV CP monomer (PDB code 1EI7) and the N-His-TMV CP^19^ monomer. The shifting N-His-TMV CP^19^ LR cylindrical helix is highlighted by an arrow. (C) Structure comparison between the TMV CP monomer (PDB code 3JO6) and the N-His-TMV CP^19^ monomer. The shifting N-His-TMV CP^19^ LR cylindrical helix is highlighted by an arrow. (D) Alignment of the side view of the TMV CP monomer (PDB code 1EI7) and the N-His-TMV CP^19^ monomer to show the different conformations in the main- and side-chain residues. The key residues in oligomer assembly are highlighted and labeled. (E) Alignment of the side view of the TMV CP monomer (PDB code 3JO6) and the N-His-TMV CP^19^ monomer to show the different conformations in the main- and side-chain residues. The key residues in oligomer assembly are highlighted and labeled.

Atoms of the inner loop region (residues 90–110) and the last four residues (154–158) had high B factors both in the previously reported disk structure (PDB code: 1EI7) and helical structure (PDB code: 3JO6), implying that these regions were disordered compared with the average structure [12], [19]. After truncating the last four residues (154–158) and introducing a six-His tag at the TMV CP N-terminus, the modified TMV CP, N-His-TMV CP^19^, had a higher atomic B factor near the missing region (LR region residues 104–111 of the a-ring; residues 107–111 of the b-ring), indicating a poorly ordered structure in the LR region; the N-His-TMV CP^19^ had a lower atomic B factor in the central helix (right slewed helical residues 38–51 and right radial helical residues 76–87) and the C-terminal loop region (residues 148–154), indicating a more tightly packed, well-ordered structure in these regions (**Figure 8A** **,** **Figure 8B** **and** **Figure 8C**). For the N-His-TMV CP^19^, the poorly ordered structure was stabilized by protein–protein hydrogen-bonding interactions in the adjacent subunit, such as Asn25-Ser15, Tyr72-Thr28, Phe35-Asp88, Gln36-Asp88 and Arg122-Asp88. These interactions resulted in conformational changes of residues, such as Gln36 and Asp115 in the LR region (**Figure 8D** **and** **Figure 8E**), which differ from the previously reported disk structure (PDB code: 1EI7) and helical structure (PDB code: 3JO6). The close-up electron density map and fitted coordinates between N-His-TMV CP^19^ and the previously reported disk structures are described in **Figure S11 and** **Figure S12**. In addition, the direct protein–protein interactions by the salt-bridges and hydrogen-bonding networks between layers of the N-His-TMV CP^19^ structure were different from the previously reported disk structure (PDB code: 1EI7) and helical structure (PDB code: 3JO6), and these interactions could be interpreted as conformational changes affected by Thr59 and Arg134, as shown in **Figure 8D** **and** **Figure 8E**. A cross-section of the X-ray map and fitted coordinates of the HR residues and LR loop residues of N-His-TMV CP_19_ and the previously reported TMV CP disk (PDB code 1EI7) are provided in **Figure S15**, the N-His-TMV CP^19^ residues (Ser1, Tyr2, Val58, Pro63, Asp64, Thr89, Arg90, Asn91, Arg141, Trp152, Thr153, Ser154) of the HR and the LR loop regions are different from those residues in the previous structure. To summarize, the main differences between N-His-TMV CP^19^ and TMV CP disks involve trans-layers proteins.

## Conclusions

Full-length N-His-TMV CP^19^ is easily overexpressed and purified to large scale; however, it is difficult to crystallize. After truncating four residues at the N-His-TMV CP^19^ C-terminus, high-quality crystals of N-His-TMV CP^19^ were easily obtained by hanging-drop vapor diffusion and micro-seeding methods. This phenomenon shows the conformational flexibility of the four residues at the C-terminus, which affects the crystal packing. The structure of N-His-TMV CP^19^ showed that the HR loop region (residues148–154) at the C-terminus had a low atomic B factor, which indicates a well-ordered structure in the HR loop region. Compared with the helical and disk structures of the previously reported TMV CP, the N-His-TMV CP^19^ subunits displayed different conformations in the LR and HR regions, and its four-layer aggregate disk was mainly retained by protein–protein salt-bridges and hydrogen-bonding networks between the trans-layers. In addition, the N-His-TMV CP^19^ could package RNA effectively and could assemble into the infectious virus particle with RNA. Thus, the N-His-TMV CP^19^ protein can form a reassembled disk or helical structure, and this structure exists in an RNA base-binding pocket. This pocket can be stabilized by TMV RNA and reassembled into a complete virus, and thus can serve as a potential molecular target of anti-plant virus drugs. These principles can also be applied in biology and structure-based drug design.

## Materials and Methods

### Isolation of TMV RNA and Construction of TMV CPs

TMV (common strain) was isolated from infected *Nicotiana tabacum* K_326_ leaves cultivated in a greenhouse at the Center for Research and Development of Fine Chemicals of Guizhou University. The virus was purified using the method described by Gooding [27], [28] and modified by Shire [29]. TMV RNA was extracted from purified virus by treatment with phenol and sodium dodecyl sulfate [30], [31]. The full-length viral cDNA sequence was generated by reverse transcription of TMV RNA using primer 1 (5′-GGAATTCCATATGTCTTACAGTATCACTACTCC-3′, the restriction site is underlined) in 50 mM Tris-HCl (pH 8.0), 8.0 mM magnesium chloride, 75 mM potassium chloride, 10 mM DL-dithiothreitol, 1.0 mM dNTPs, 0.5 unit/μL, AMV reverse transcriptase (TaKaRa), and 1.0 unit/μL RNase inhibitor (TaKaRa) for 1.5 hr at 85°C. The N-His-TMV CP^19^ gene was amplified by PCR using viral cDNA as template, with primer 1 and primer 9 (5′-CCGCTCGAGTCAAGAGGTCCAAACCAAACC-3′, the restriction site is underlined).

### N-His-TMV CP^19^ Protein Expression and Purification

A six-His tag was introduced at the N-terminus to enable protein purification [25], and four amino acids (Gly155, Pro156, Ala157 and Thr158) were deleted at the C-terminus of WT TMV CP for crystallization [24]. The N-His-TMV CP^19^ protein was constructed by cloning common strains of the TMV CP gene into the *Nde I* and *Xho I* sites of pET28a vector (Novagen) and was expressed in *E. coli* BL_21_ (DE_3_) RIL cells (Novagen). The cells were grown in Luria-Bertani medium supplemented with 50 μg/mL kanamycin at 37°C to an OD_600_ of 0.65, shaking at 220 rpm. Then, the expression of protein was induced with 0.1 mM isopropyl-1-thio-*β*-D-galactopyranoside (IPTG) at 16°C overnight. The cells were harvested by centrifugation and then stored at −80°C. Cell pellets were resuspended in lysis buffer (30 mM PB, 300 mM NaCl, 1 mM *β*-mercaptoethanol, pH 8.0) and then lysed at 4°C by sonication. After centrifugation at 15,000 g for 30 min to remove any insoluble material, the supernatant was loaded onto a 5 mL HisTrap High-Performance column (GE Healthcare), and the protein was eluted with a single gradient of 50 mM to 400 mM imidazole (pH 8.0). The C-terminally truncated protein was further purified by gel filtration (SEC) using a superdex 200 column (GE Healthcare, 120 mL) in a buffer containing 20 mM PB (pH 8.0) and 100 mM NaCl. Gel filtration was performed at 4°C using a calibrated superdex 200 10/300 GL column (GE Healthcare) attached to an AKTA purifier fast protein liquid chromatography system (GE Healthcare). The column was equilibrated with a buffer containing 10 mM PB (pH 7.2) or 100 mM NaCl depending on the TMV CP disk buffer [32]. Molecular mass standards (Bio-Rad) included thyroglobulin (669 kDa), ferritin (440 kDa), bovine serum albumin (67 kDa), *β*-lactoglobulin (35 kDa), ribonuclease A (13.7 kDa), cytochrome (13.6 kDa), aprotinin (6.51 kDa), and vitamin B12 (1.36 kDa). The proteins were monitored by absorbance at a wavelength of 280 nm. Native Polyacrylamide Gel Electrophoresis (PAGE) was performed on ice with the N-His-TMV CP^19^ samples, which were equilibrated overnight in a buffer containing 10 mM PB (pH 7.2) and 100 mM NaCl. A 20 µL sample was treated with 20 µL 2× loading buffer [12.5% 0.5 M Tris-HCl (V/V), pH 6.8, 0.5% bromophenol blue (W/V) and 30% glycerin (V/V)], and then, 8 μL of sample and 4 μL of protein marker were loaded onto a native-PAGE gel (4% stacking and 17% separating gel). Electrophoresis was run in 1× native page buffer (Tris-Gly, PH 8.8) at 0°C for about 1 hr [33]. After electrophoresis, the lanes were stained with coomassie blue [34], [35] to locate the protein and then destained with methanol and glacial acetic acid.

### Reconstituted N-His-TMV CP^19^ Virus

One milliliter of purified self-assembled N-His-TMV CP^19^ disk (1.8 mg/mL), incubated in 10 mM PB, 100 mM NaCl, pH 7.2 solution, was mixed with 0.2 mL purified TMV RNA (0.5 mg/mL), and then the mixture was incubated at 22°C for 20 h. Suspensions were centrifuged at 5,000 rpm for 1 min, and then the reconstituted virus was obtained [4], [8], [29]. A 20 μL portion of protein solution was deposited onto a 300-mesh formvar-carbon-coated copper grid for 2 min, followed by rinsing with ddH_2_O. The grid was then stained with 20 μL of 2% aqueous solution of tungstophosphoric acid for 90 s as a negative stain [35]–[40]. Images were obtained at the Zunyi Medical University Electron Microscope Lab using a Hitachi H-7650 transmission electron microscope (TEM) with 80 kV accelerating voltage. The pattern of RNA packaging is very important for the reconstitution of infectious virus, which has a similar mechanism to other species [41]–[43]. Growing leaves of *N. glutinosa* were mechanically inoculated with reconstructed virus or the WT virus as a control. The local lesion numbers were recorded 3−4 days after inoculation.

### Crystallization of N-His-TMV CP^19^

Crystallization was performed at 22°C by using the hanging-drop vapor diffusion method. N-His-TMV CP^19^ protein was concentrated to 14.0 mg/mL in a buffer containing 20 mM PB, pH 8.0, 100 mM NaCl, and 1 mM DTT, and then crystallization conditions were carefully screened [24]. Briefly, 1 µL protein solution was mixed with 1 µL reservoir solution (300 mM ammonium sulfate, 100 mM Tris-HCl, pH 7.7) and equilibrated against 0.6 mL reservoir solution. The micro-crystals were grown for 3 days and collected and deposited with a seeding tool (Hampton Research) [23]. Optimization of crystallization was performed in reservoir solution at different protein concentrations, ion concentrations (100 mM to 300 mM ammonium sulfate, 100 mM Tris-HCl) and pH (7.5 to 8.5). High-quality crystals of N-His-TMV CP^19^ were mounted and flash-frozen in liquid nitrogen following cryo-protection with reservoir solution containing an additional 30% (v/v) glycerol [44].

### Data Collection and Structure Determination

Diffraction data were collected at the Shanghai Synchrotron Radiation Facility (SSRF) beamline 17U. All X-ray data were processed using the HKL2000 program [45] and converted to structure factors within the CCP4 program [46]. A typical octahedral-shaped crystal belongs to the space group P2_1_2_1_2. The structure was solved by molecular replacement in Phaser [47] using the published WT TMV CP monomer as a search model (PDB code 1EI7). The N-His-TMV CP^19^ protein model was manually built in COOT [48], and computational refinement was conducted with the program REFMAC5 [49] in the CCP4 suite. Molecular graphic figures were prepared in PyMOL [50]. The local noncrystallographic symmetry (NCS) axis was refined by a grid search and varied orientation or translation of the asymmetrical unit was done using XPLOR rigid body dynamics. After using the NCS restraints option of the XPLOR refinement program [51], the 34 subunits of the asymmetrical unit were refined individually using simulated annealing. There were no departures from the noncrystallographic symmetry found during this procedure of refinement, as indicated by the RMSD between the individual subunits (0.01–0.02 Å) [12].

## Supporting Information

Figure S1 **Ramachandran Plot of the N-His-TMV CP_19_** **monomer.**
(TIF)

Figure S2 **Comparison of the structure of the truncated TMV CP and previous structures by pyMOL.** The following structural comparisons are shown: (A) The N-His-TMV CP^19^ a-chain and the TMV CP (PDB code: 1EI7) a-chain; (B) The N-His-TMV CP^19^ a-chain and the TMV CP (PDB code: 1EI7) b-chain; (C) The N-His-TMV CP^19^ a-chain and the TMV CP (PDB code: 3JO6) a-chain; (D) The N-His-TMV CP^19^ a- and b-chains and TMV CP (PDB code: 1EI7) a- and b-chains.
(TIF)

Figure S3 **Superimposition of the N-His-TMV CP^19^ a- and b-chains by pyMOL.** (A) Carbon-alpha trace showing a superimposition of the N-His-TMV CP^19^ a- and b-chains; (B) Bonds showing the superimposition of the N-His-TMV CP^19^ a- and b-chains.
(TIF)

Figure S4 **Electron density map of the N-His-TMV CP^19^ by Coot.** (A) The electron density map of N-His-TMV CP^19^viewed looking perpendicular to the crystallographic twofold axis, (B) An overview of the electron density map of N-His-TMV CP^19^ is provided at different angles.
(TIF)

Figure S5 **A well-defined electron density map of the N-His-TMV CP^19^ monomer by Coot.** (A) Carbon-alpha trace showing a well-defined electron density map of the N-His-TMV CP^19^ monomer; (B) Bonds showing a well-defined electron density map of the N-His-TMV CP^19^ monomer.
(TIF)

Figure S6 **Electron microscopic analysis of tobacco leaves infected with reconstituted particles.** (A) Original image of the tobacco leaves infected with reconstituted particles. (B) Magnified view of the tobacco leaves infected with reconstituted particles. N-His-TMV CP^19^ discs are enclosed in red boxes.
(TIF)

Figure S7 **Assembly of the four-layer aggregate disks of N-His-TMV CP^19^ and wild type TMV CP as assessed by native-PAGE and SEC.** (A) Disks of N-His-TMV CP^19^ eluted at 7.9 mL on SEC. (B) Disks of N-His-TMV CP^19^ were analyzed by native-PAGE. (C) Disks of wild TMV CP eluted at 7.9 mL on SEC. (D) Disks of wild TMV CP were analyzed by native-PAGE.
(TIF)

Figure S8 **X-ray density map and fitted coordinates of a cross-section corresponding to the HR residues and LR loop residues of N-His-TMV CP^19^.** (A) Cross-section of an X-ray map and fitted coordinates at the HR residues of N-His-TMV CP_19_. (B) Cross-section of an X-ray map and fitted coordinates at the LR loop residues of N-His-TMV CP_19_.
(TIF)

Figure S9 **Side chain density map and fitted coordinates corresponding to the side chain residues of N-His-TMV CP^19^.**
(TIF)

Figure S10 **Electron density map and fitted coordinates corresponding to the inter-chain interactions within the N-His-TMV CP^19^ b-ring subunit.** The electron density map and fitted coordinates corresponding to the inter-chain interactions between the following residues in the N-His-TMV CP^19^ disk of the b-ring are shown: (A) Phe35-Asp88 and Gln36-Asp88. (B) Tyr72-Thr28. (C) Arg113-Asp115. (D) Asn25-Ser15.
(TIF)

Figure S11 **Close-up electron density map and fitted coordinates showing the side chain residues of N-His-TMV CP^19^ and the reported TMV CP (PDB code 1EI7) subunits from** **Figure 8D** **.**
(TIF)

Figure S12 **Close-up electron density map and fitted coordinates showing the side chain residues of N-His-TMV CP^19^ and the reported TMV CP (PDB code 3JO6) subunits from** **Figure 8E** **.**
(TIF)

Figure S13 **Electron density map and fitted coordinates corresponding to the protein–protein interactions of the N-His-TMV CP^19^ trans-layer.** The electron density map and fitted coordinates between the following N-His-TMV CP^19^ protein–protein interactions are shown: (A) The Asp19-Arg134 and Asp66-Arg134 between the b-pair and a-pair. (**B**) The Asp19-Arg134 and Asp66-Arg134 between the b-pair and a-pair and the Asp19-Arg134 and Asp66-Arg134 between the previously reported TMV CP disk structures (PDB code 1EI7). In the previously reported TMV CP disk structure, the protein–protein interactions involved in Asp19-Arg134 and Asp66-Arg134 between the b-pairs and a-pairs were mediated by water. (**C**) The Thr59-Thr59 between the a-pair and a-pair. (**D**) The Lys53-Glu22 between the a-pair and a-pair.
(TIF)

Figure S14 **Close-up electron density map and fitted coordinates showing the inter-chain residues of N-His-TMV CP^19^ and the previously reported TMV CP (PDB code 1EI7) subunits.**
(TIF)

Figure S15 **Cross-section of the X-ray map and fitted coordinates in the HR residues and LR loop residues of N-His-TMV CP^19^ and the previously reported TMV CP disk (PDB code 1EI7).** Cross-section of the X-ray map and fitted coordinates of N-His-TMV CP^19^ and the TMV CP disk structure (PDB code 1EI7) are shown for the region of: (A) the HR residues; and (B) the LR loop residues.
(TIF)

Table S1 **Data collection and refinement statistics.**
(DOCX)
