# Supplementary figures and images for: Crystal Structure of a Four-Layer Aggregate of Engineered TMV CP Implies the Importance of Terminal Residues for Oligomer Assembly
Source: PLoS One. 2013 Nov 4;8(11):e77717. doi: 10.1371/journal.pone.0077717 (PMC3817195; doi:10.1371/journal.pone.0077717)

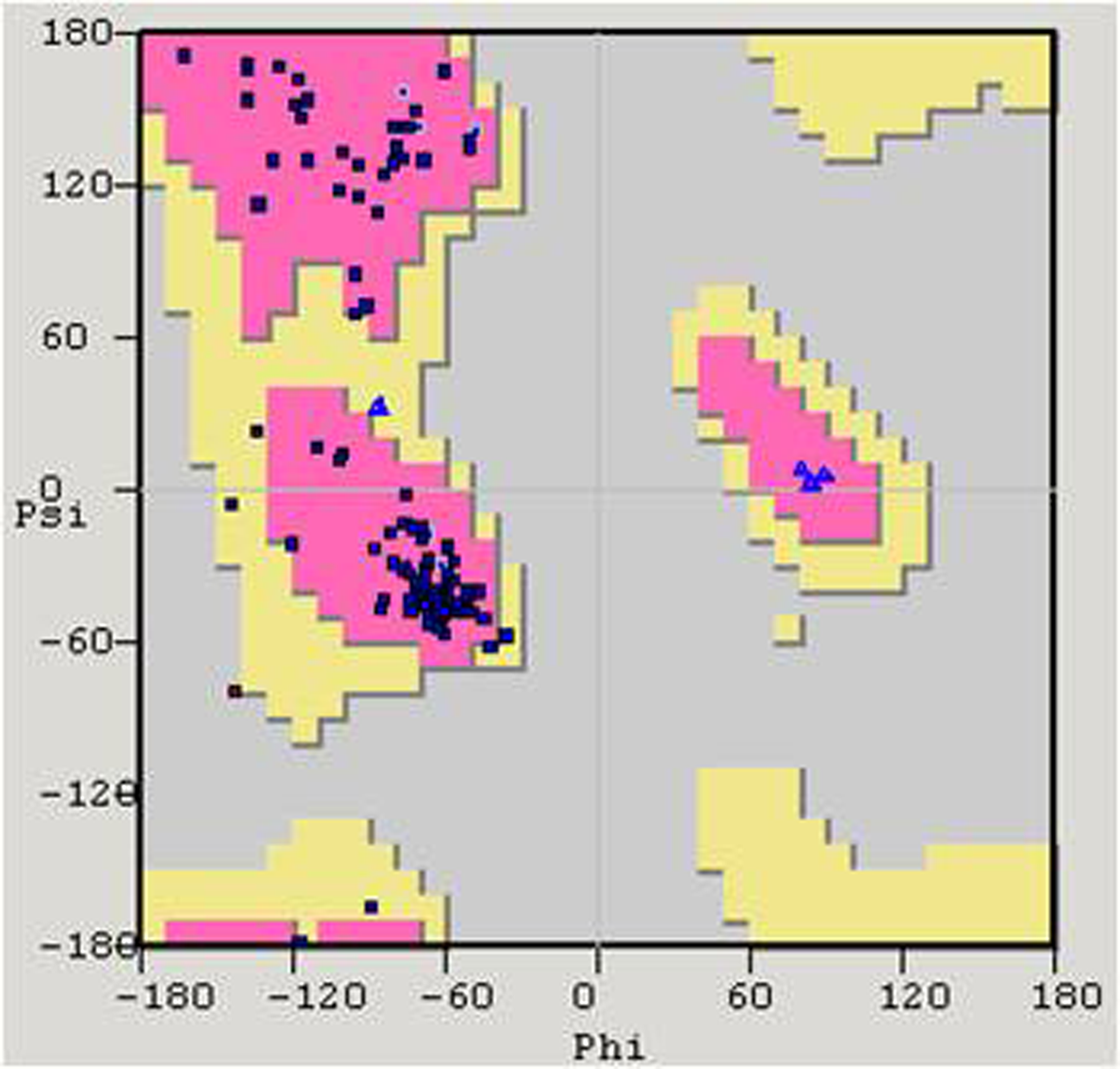

Supplement: Figure S1 — Ramachandran Plot of the N-His-TMV CP19 monomer. (TIF) [file pone.0077717.s001.tif]

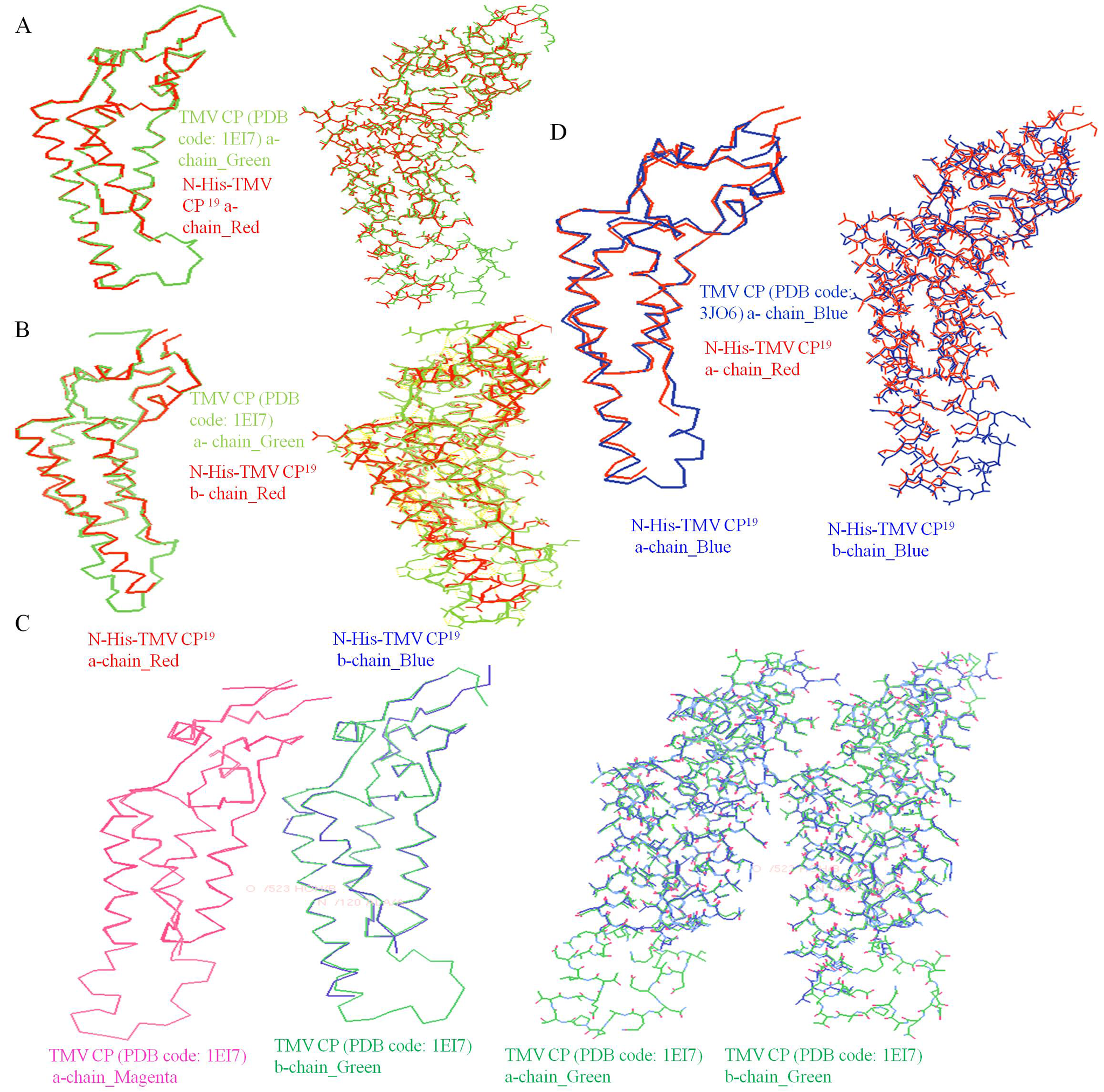

Supplement: Figure S2 — Comparison of the structure of the truncated TMV CP and previous structures by pyMOL. The following structural comparisons are shown: (A) The N-His-TMV CP19 a-chain and the TMV CP (PDB code: 1EI7) a-chain; (B) The N-His-TMV CP19 a-chain and the TMV CP (PDB code: 1EI7) b-chain; (C) The N-His-TMV CP19 a-chain and the TMV CP (PDB code: 3JO6) a-chain; (D) The N-His-TMV CP19 a- and b-chains and TMV CP (PDB code: 1EI7) a- and b-chains. (TIF) [file pone.0077717.s002.tif]

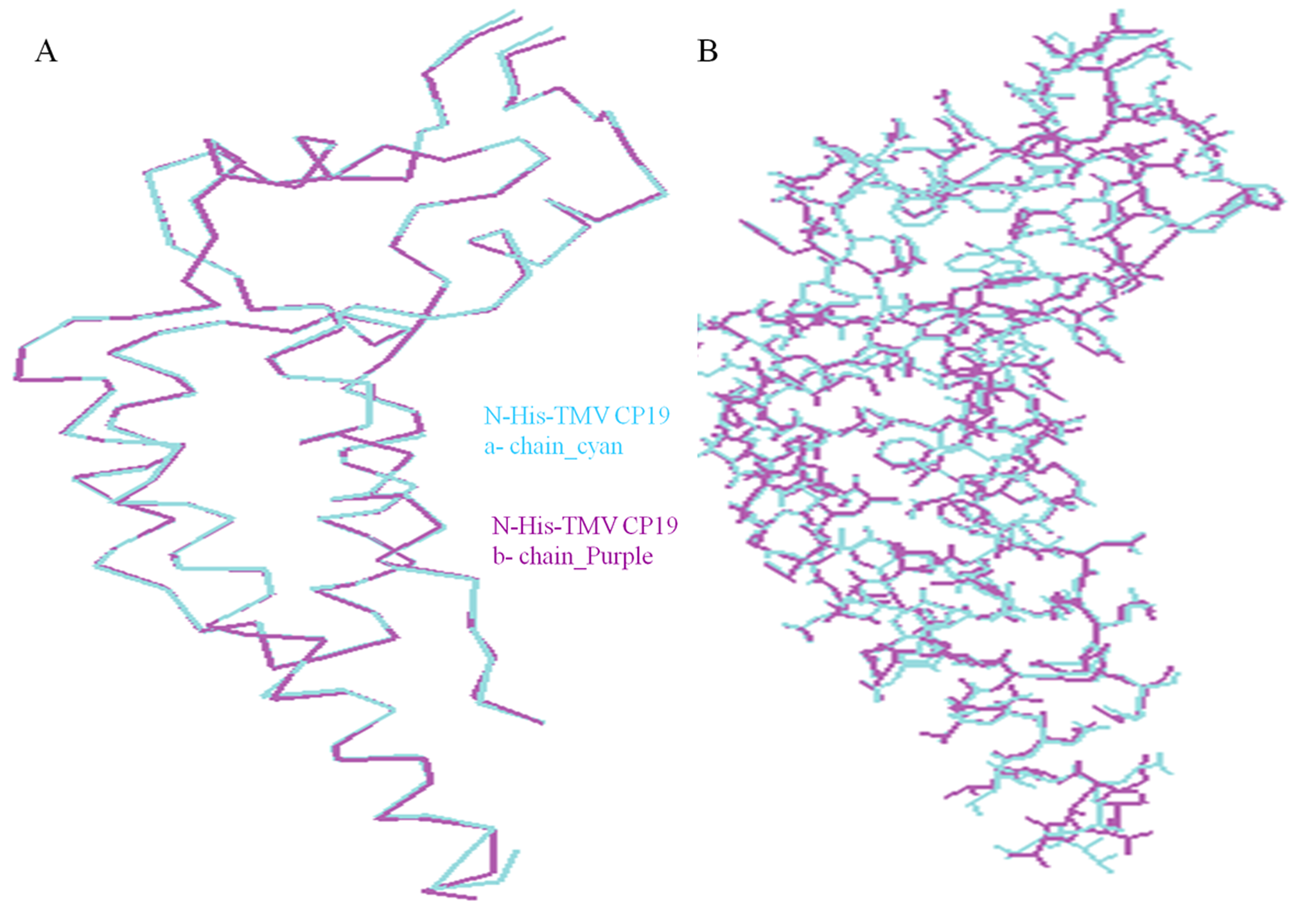

Supplement: Figure S3 — Superimposition of the N-His-TMV CP19 a- and b-chains by pyMOL. (A) Carbon-alpha trace showing a superimposition of the N-His-TMV CP19 a- and b-chains; (B) Bonds showing the superimposition of the N-His-TMV CP19 a- and b-chains. (TIF) [file pone.0077717.s003.tif]

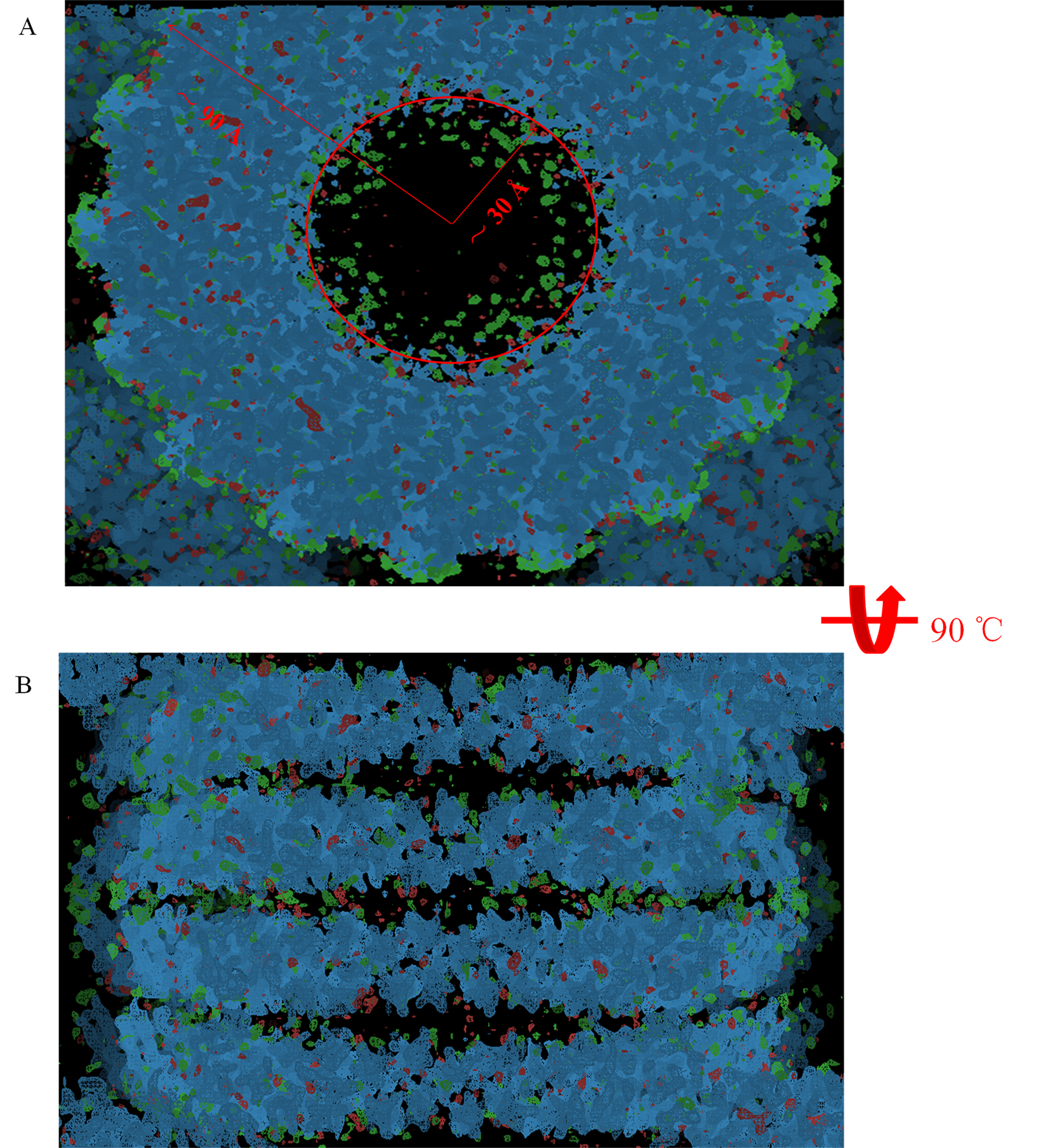

Supplement: Figure S4 — Electron density map of the N-His-TMV CP19 by Coot. (A) The electron density map of N-His-TMV CP19viewed looking perpendicular to the crystallographic twofold axis, (B) An overview of the electron density map of N-His-TMV CP19 is provided at different angles. (TIF) [file pone.0077717.s004.tif]

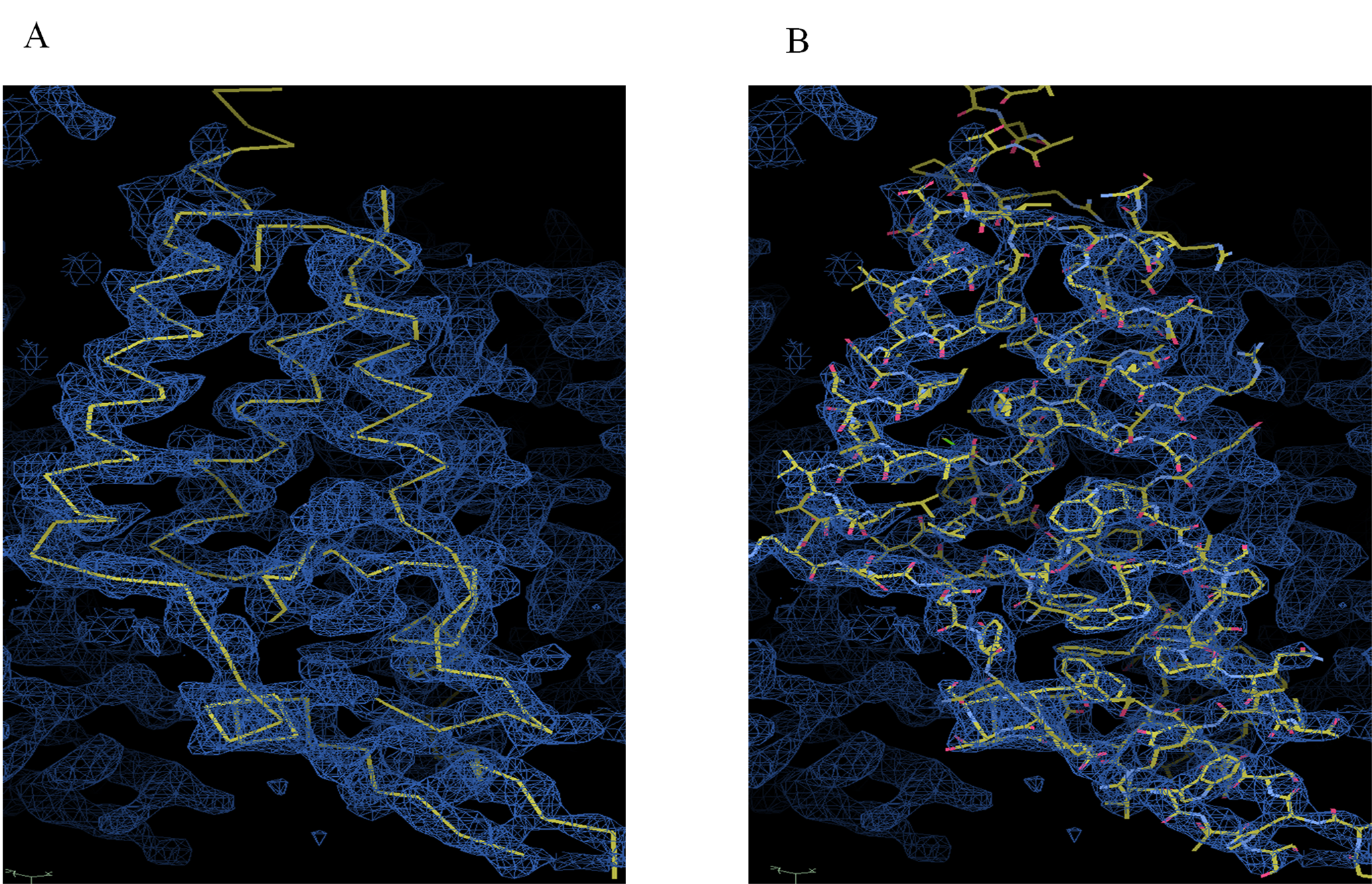

Supplement: Figure S5 — A well-defined electron density map of the N-His-TMV CP19 monomer by Coot. (A) Carbon-alpha trace showing a well-defined electron density map of the N-His-TMV CP19 monomer; (B) Bonds showing a well-defined electron density map of the N-His-TMV CP19 monomer. (TIF) [file pone.0077717.s005.tif]

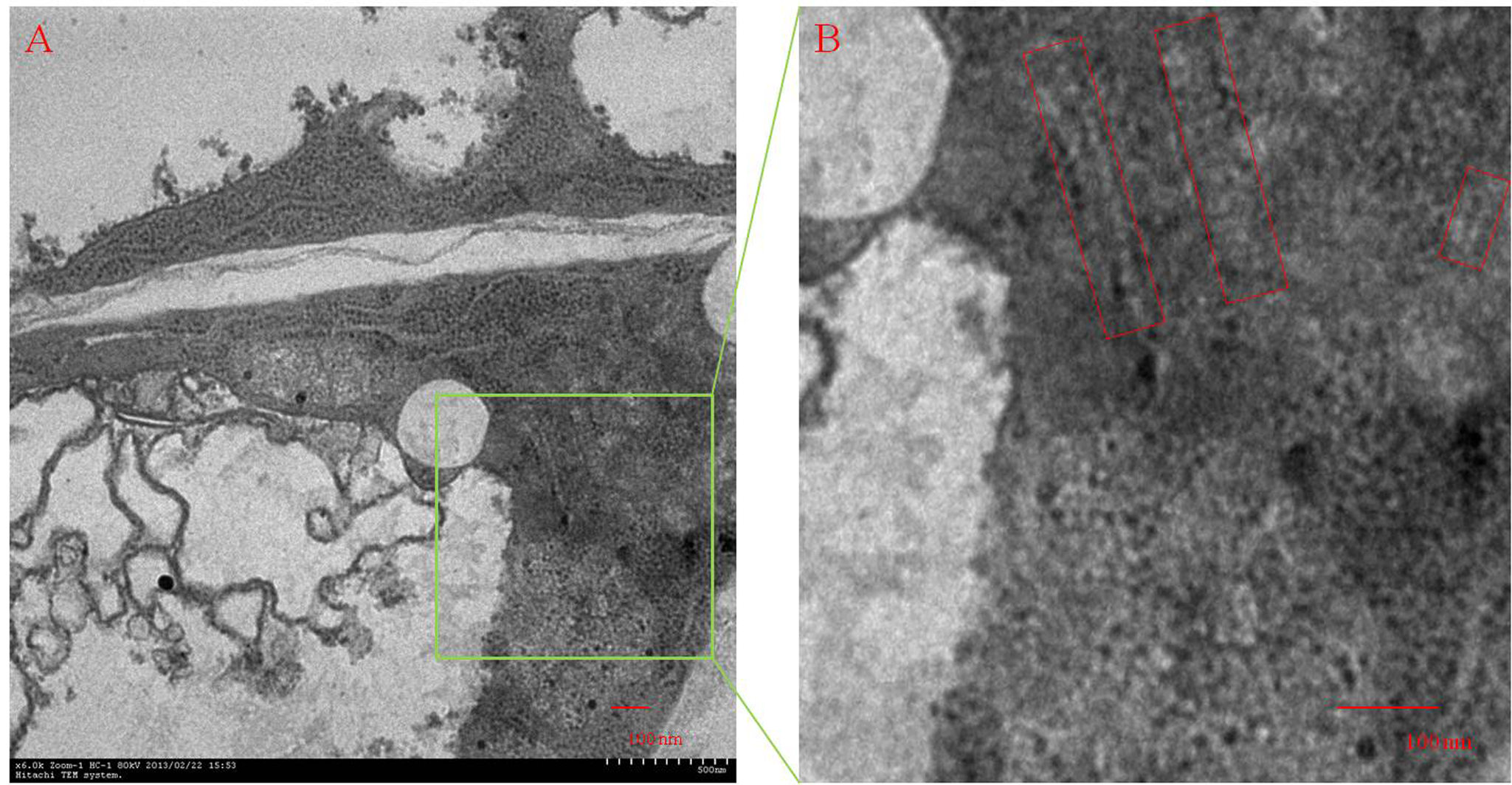

Supplement: Figure S6 — Electron microscopic analysis of tobacco leaves infected with reconstituted particles. (A) Original image of the tobacco leaves infected with reconstituted particles. (B) Magnified view of the tobacco leaves infected with reconstituted particles. N-His-TMV CP19 discs are enclosed in red boxes. (TIF) [file pone.0077717.s006.tif]

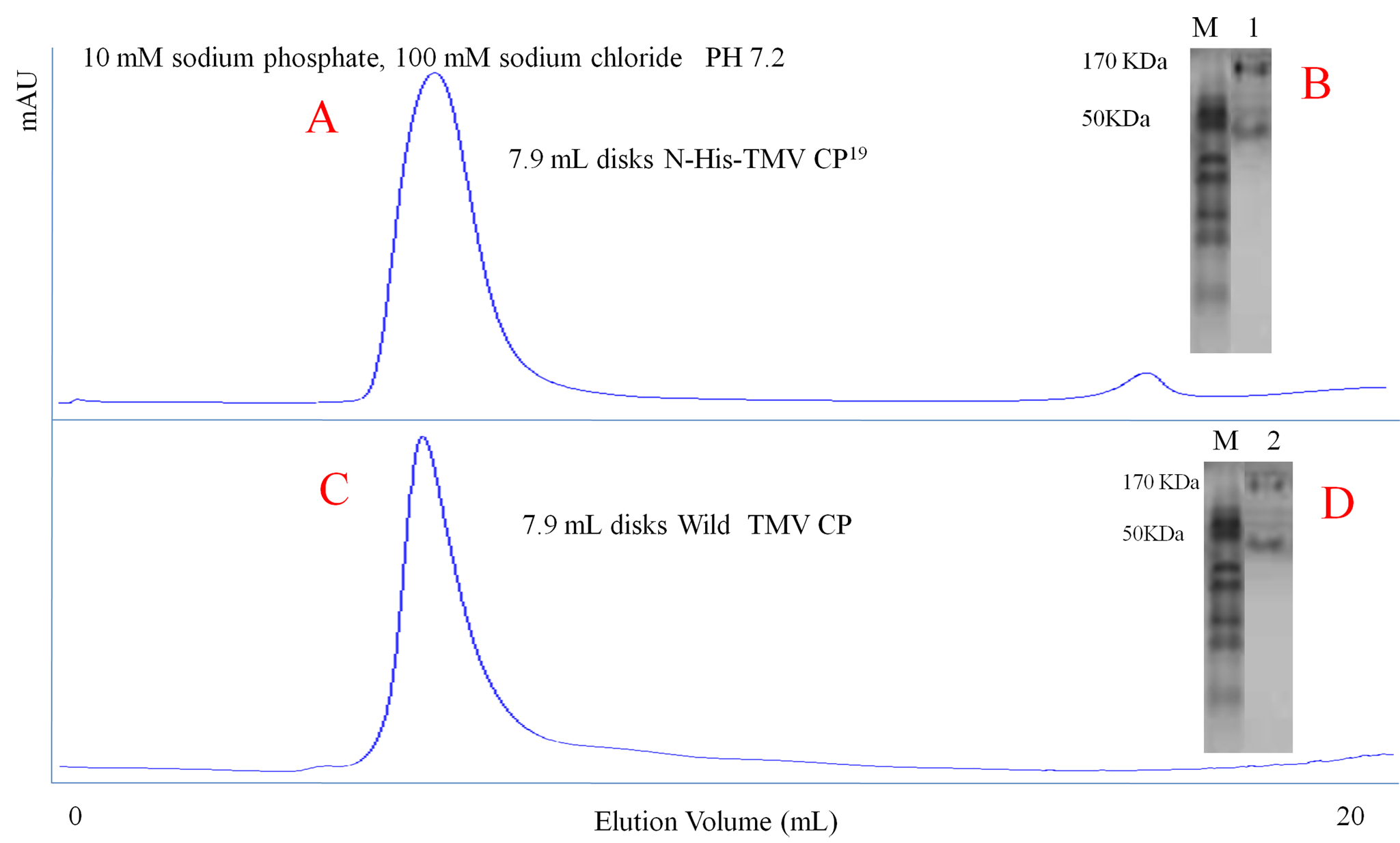

Supplement: Figure S7 — Assembly of the four-layer aggregate disks of N-His-TMV CP19 and wild type TMV CP as assessed by native-PAGE and SEC. (A) Disks of N-His-TMV CP19 eluted at 7.9 mL on SEC. (B) Disks of N-His-TMV CP19 were analyzed by native-PAGE. (C) Disks of wild TMV CP eluted at 7.9 mL on SEC. (D) Disks of wild TMV CP were analyzed by native-PAGE. (TIF) [file pone.0077717.s007.tif]

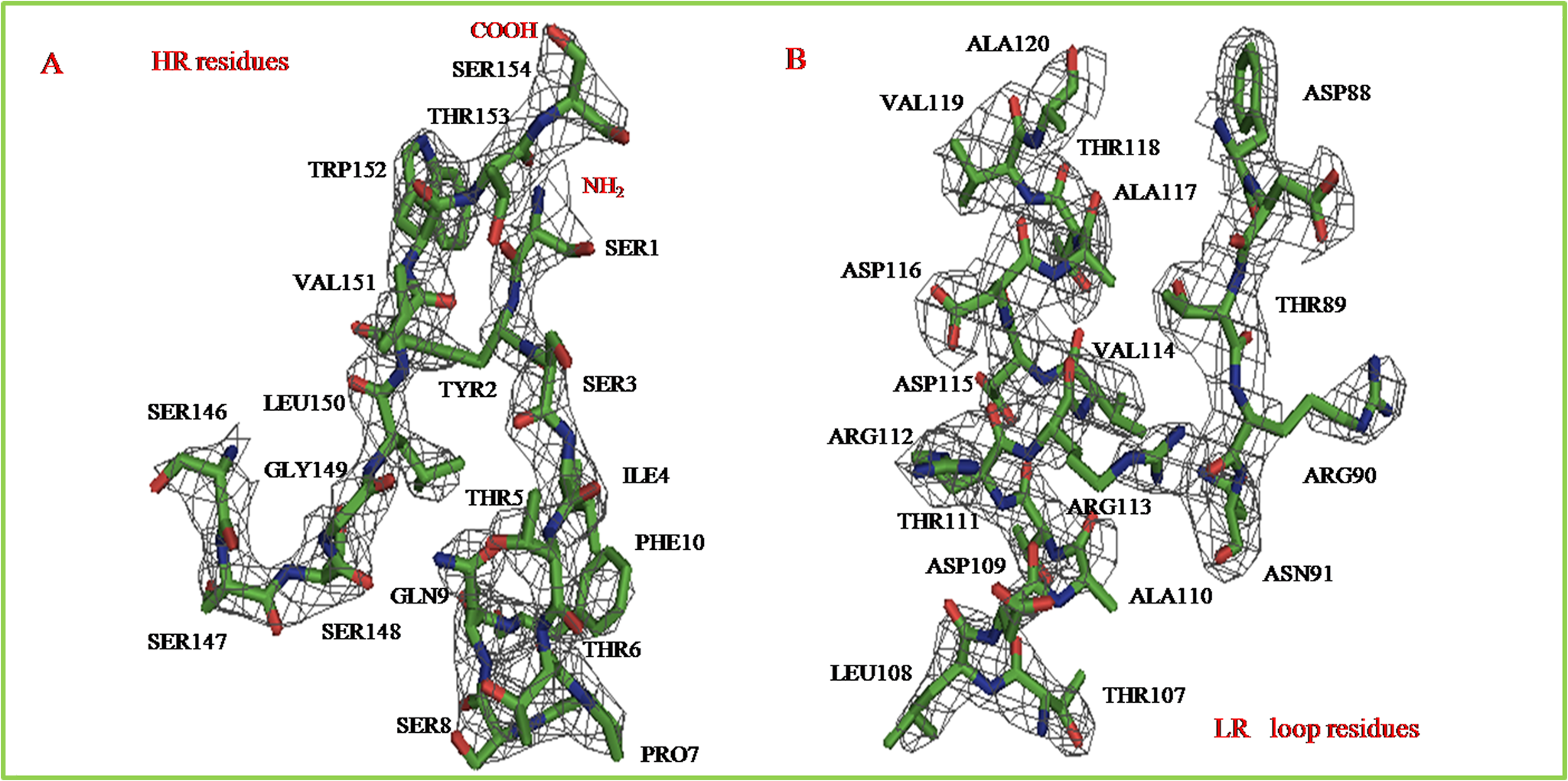

Supplement: Figure S8 — X-ray density map and fitted coordinates of a cross-section corresponding to the HR residues and LR loop residues of N-His-TMV CP19. (A) Cross-section of an X-ray map and fitted coordinates at the HR residues of N-His-TMV CP19. (B) Cross-section of an X-ray map and fitted coordinates at the LR loop residues of N-His-TMV CP19. (TIF) [file pone.0077717.s008.tif]

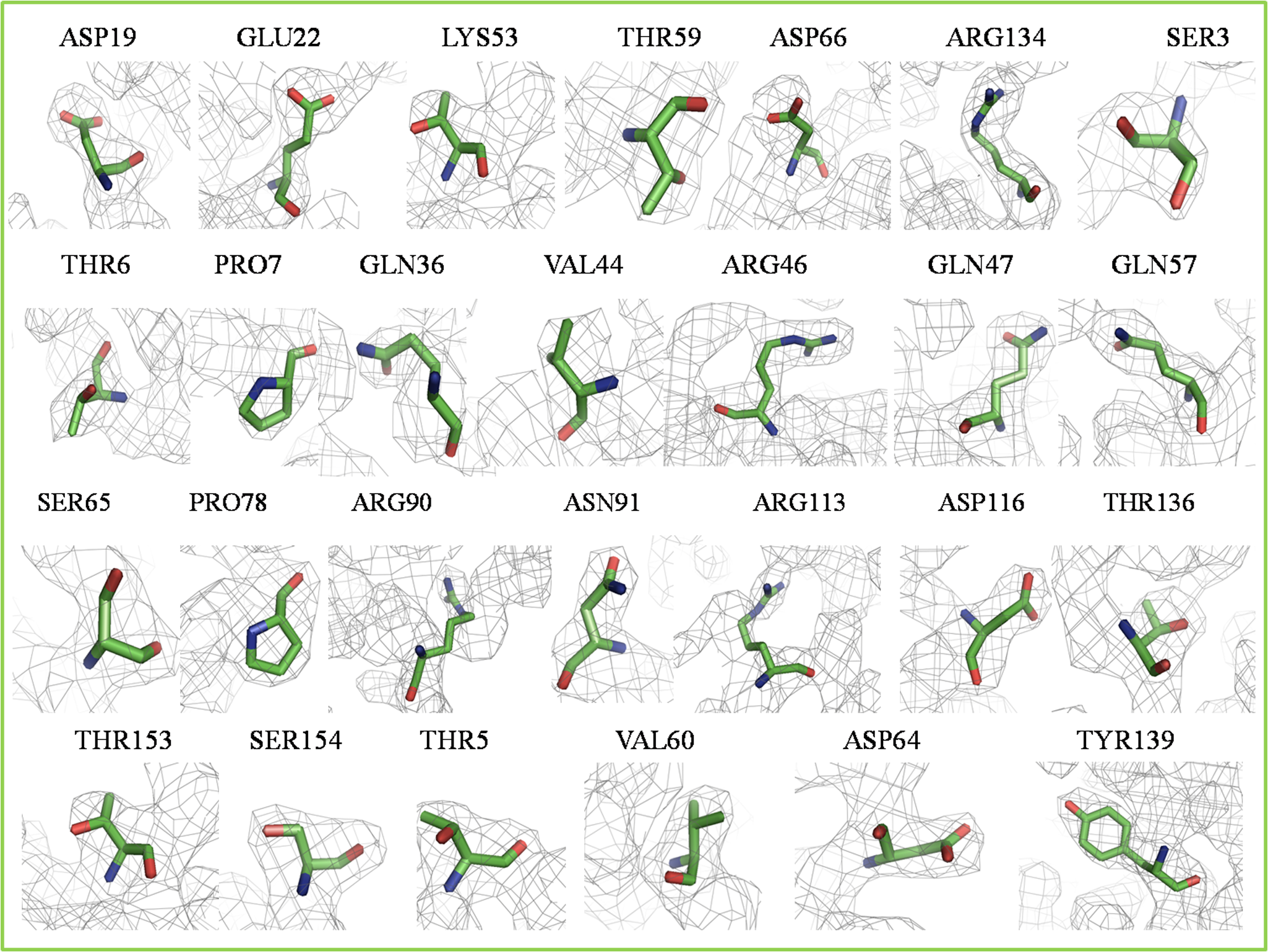

Supplement: Figure S9 — Side chain density map and fitted coordinates corresponding to the side chain residues of N-His-TMV CP19. (TIF) [file pone.0077717.s009.tif]

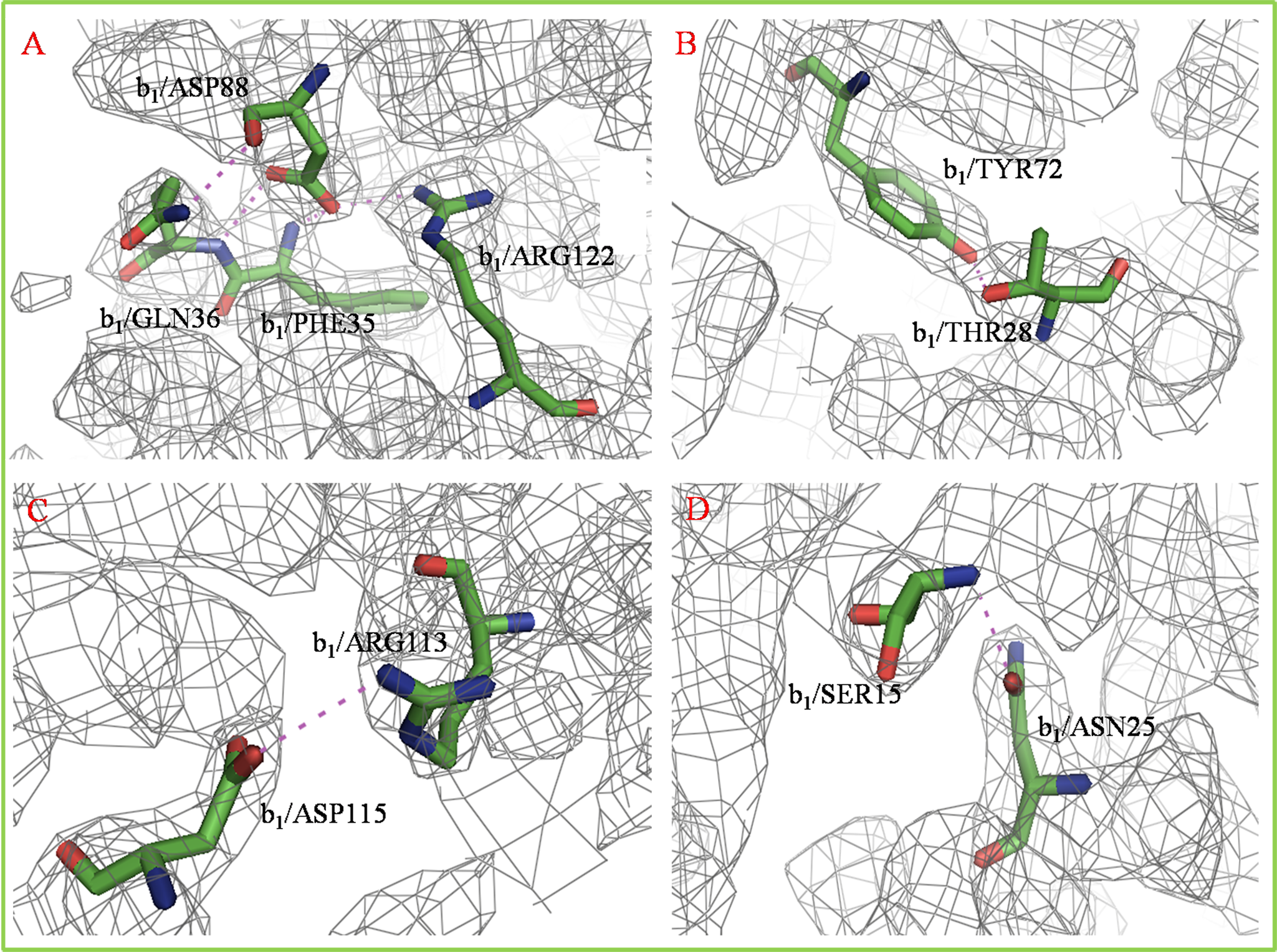

Supplement: Figure S10 — Electron density map and fitted coordinates corresponding to the inter-chain interactions within the N-His-TMV CP19 b-ring subunit. The electron density map and fitted coordinates corresponding to the inter-chain interactions between the following residues in the N-His-TMV CP19 disk of the b-ring are shown: (A) Phe35-Asp88 and Gln36-Asp88. (B) Tyr72-Thr28. (C) Arg113-Asp115. (D) Asn25-Ser15. (TIF) [file pone.0077717.s010.tif]

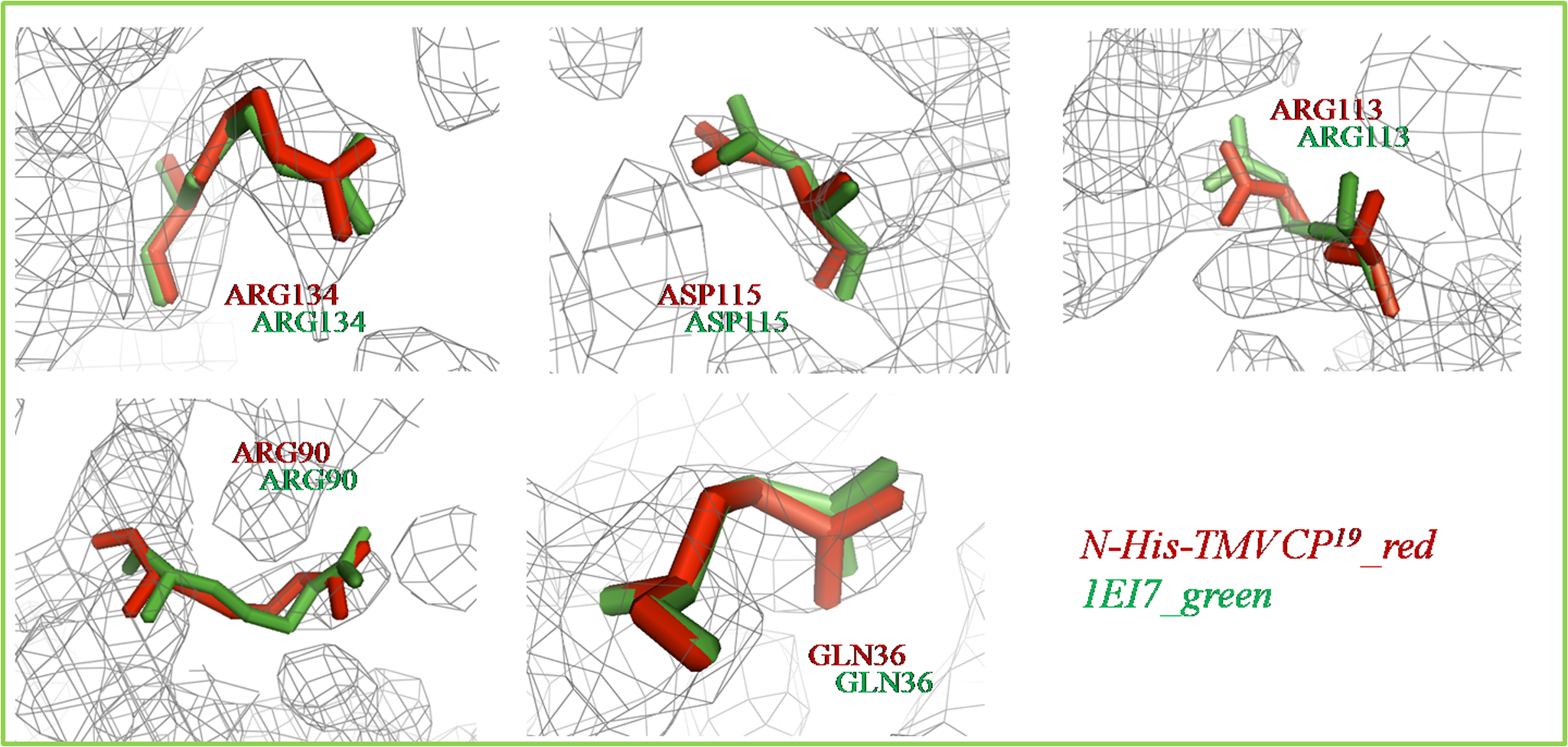

Supplement: Figure S11 — Close-up electron density map and fitted coordinates showing the side chain residues of N-His-TMV CP19 and the reported TMV CP (PDB code 1EI7) subunits from Figure 8D . (TIF) [file pone.0077717.s011.tif]

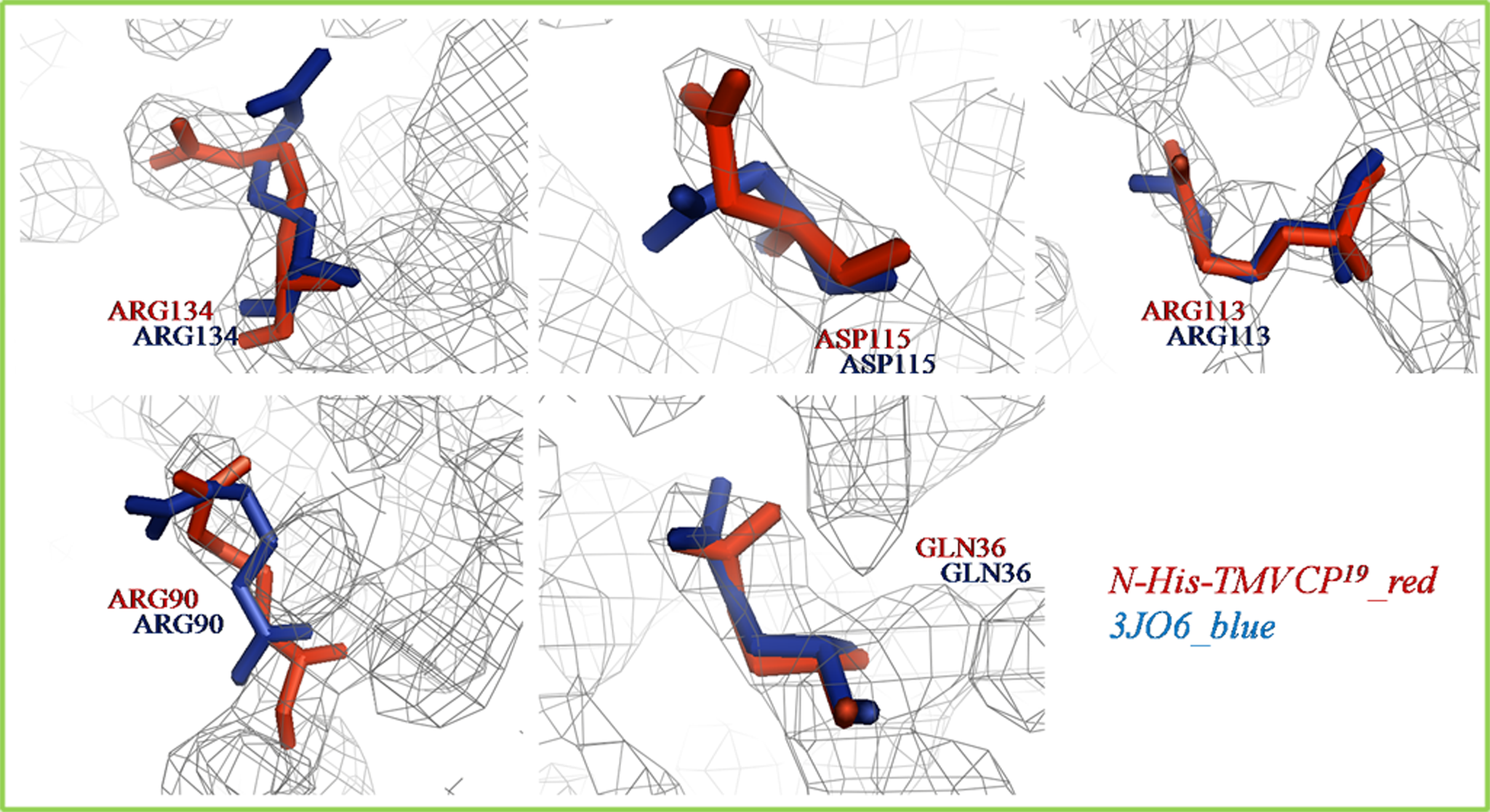

Supplement: Figure S12 — Close-up electron density map and fitted coordinates showing the side chain residues of N-His-TMV CP19 and the reported TMV CP (PDB code 3JO6) subunits from Figure 8E . (TIF) [file pone.0077717.s012.tif]

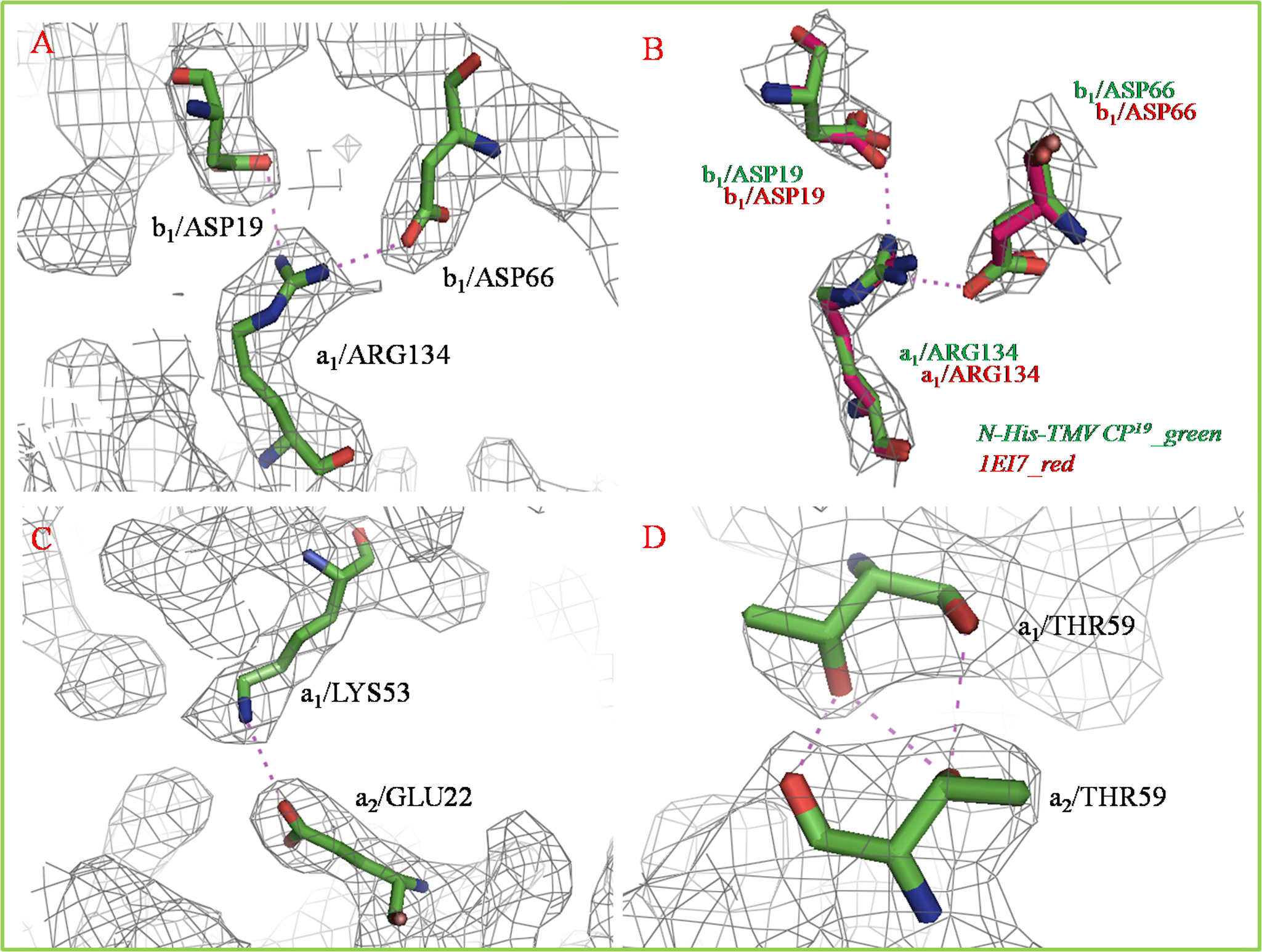

Supplement: Figure S13 — Electron density map and fitted coordinates corresponding to the protein–protein interactions of the N-His-TMV CP19 trans-layer. The electron density map and fitted coordinates between the following N-His-TMV CP19 protein–protein interactions are shown: (A) The Asp19-Arg134 and Asp66-Arg134 between the b-pair and a-pair. (B) The Asp19-Arg134 and Asp66-Arg134 between the b-pair and a-pair and the Asp19-Arg134 and Asp66-Arg134 between the previously reported TMV CP disk structures (PDB code 1EI7). In the previously reported TMV CP disk structure, the protein–protein interactions involved in Asp19-Arg134 and Asp66-Arg134 between the b-pairs and a-pairs were mediated by water. (C) The Thr59-Thr59 between the a-pair and a-pair. (D) The Lys53-Glu22 between the a-pair and a-pair. (TIF) [file pone.0077717.s013.tif]

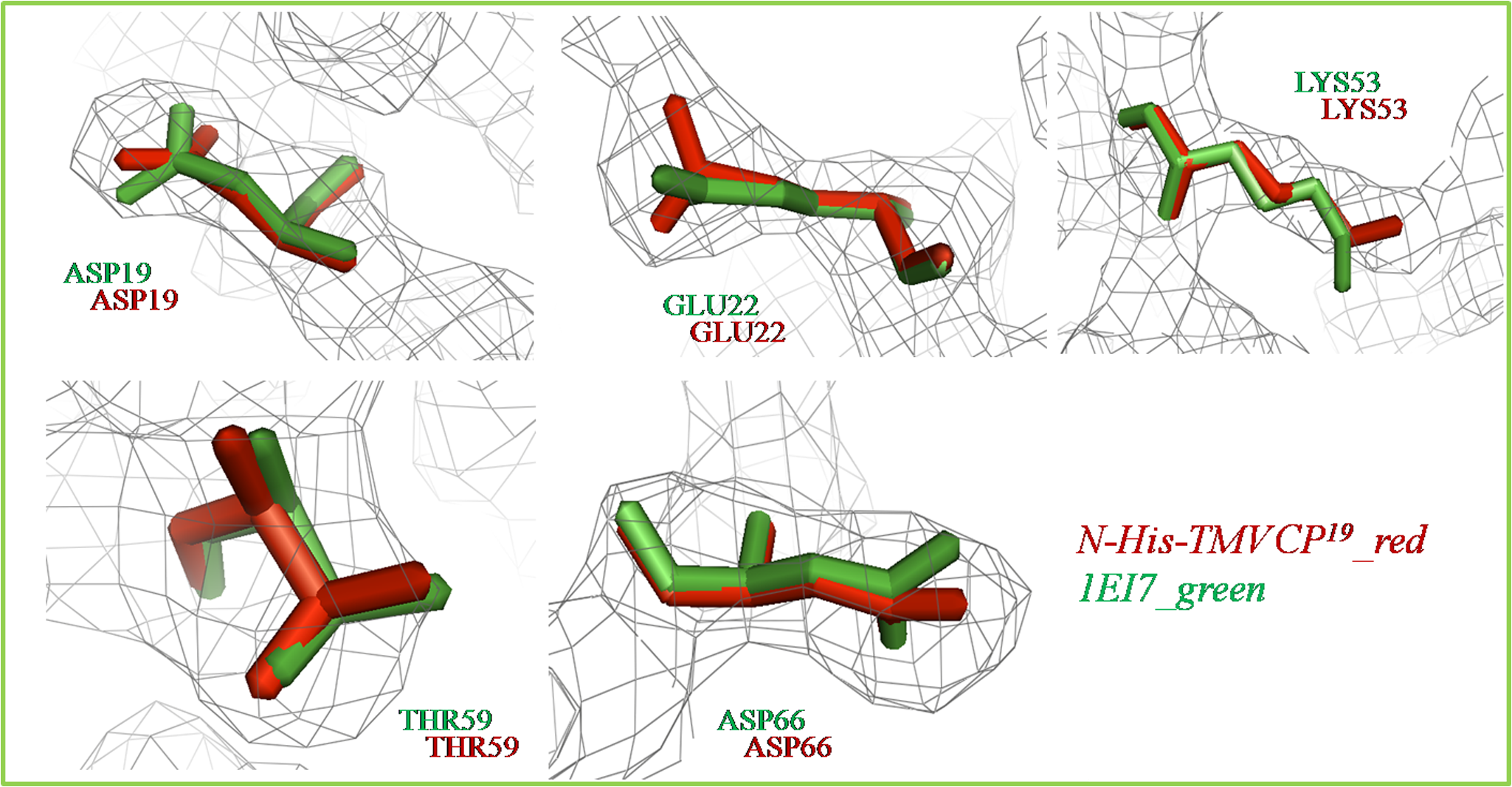

Supplement: Figure S14 — Close-up electron density map and fitted coordinates showing the inter-chain residues of N-His-TMV CP19 and the previously reported TMV CP (PDB code 1EI7) subunits. (TIF) [file pone.0077717.s014.tif]

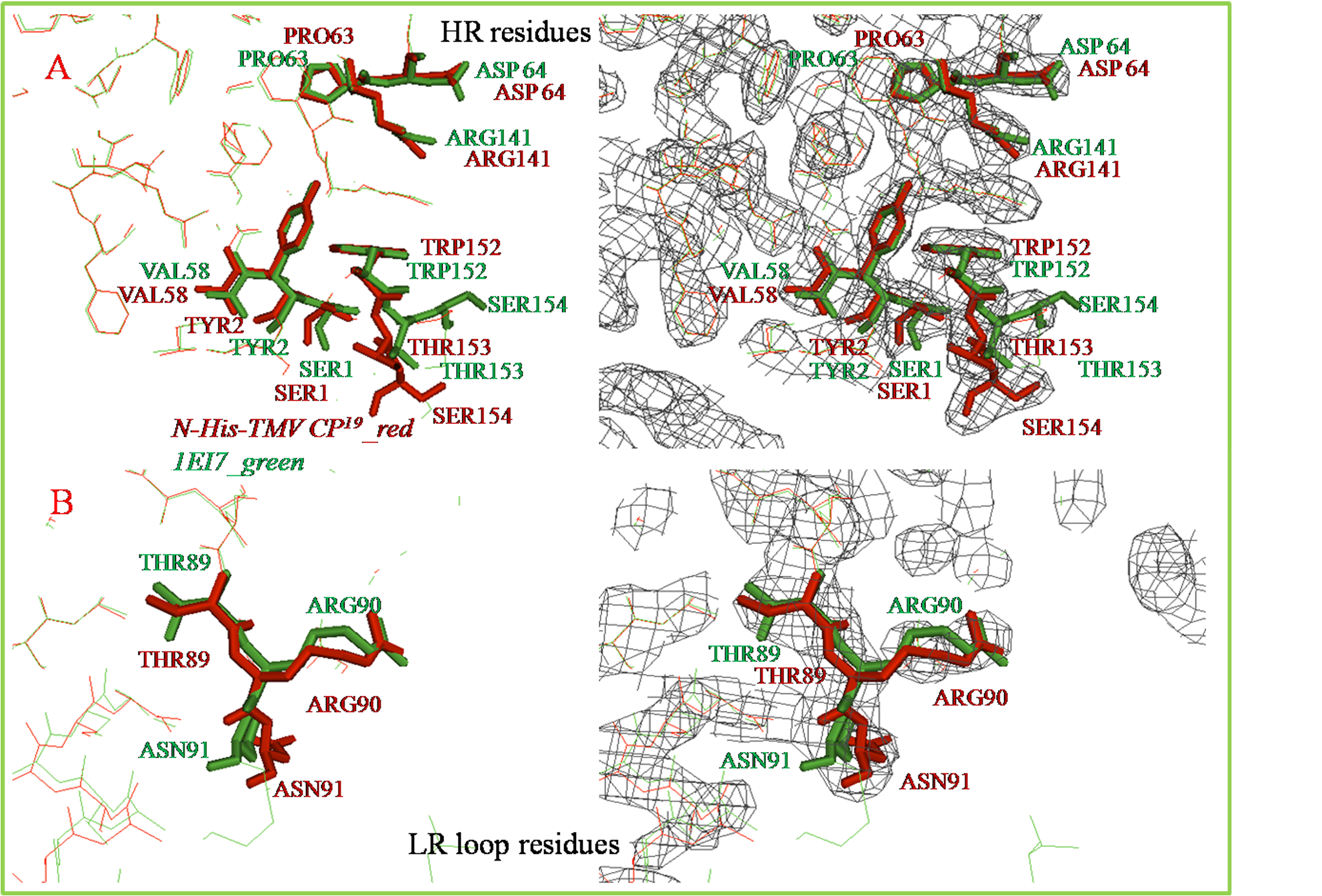

Supplement: Figure S15 — Cross-section of the X-ray map and fitted coordinates in the HR residues and LR loop residues of N-His-TMV CP19 and the previously reported TMV CP disk (PDB code 1EI7). Cross-section of the X-ray map and fitted coordinates of N-His-TMV CP19 and the TMV CP disk structure (PDB code 1EI7) are shown for the region of: (A) the HR residues; and (B) the LR loop residues. (TIF) [file pone.0077717.s015.tif]
