# Supplementary material for: Crystal Structure of a Four-Layer Aggregate of Engineered TMV CP Implies the Importance of Terminal Residues for Oligomer Assembly
Source: PLoS One. 2013 Nov 4;8(11):e77717. doi: 10.1371/journal.pone.0077717 (PMC3817195; doi:10.1371/journal.pone.0077717)
Supplement: Table S1 — Data collection and refinement statistics. (DOCX) [file pone.0077717.s016.docx]

**Table S1**

|  | N-His-TMV CP^19^ |
| --- | --- |
| **Data collection** |  |
| Space group | P2_1_2_1_2 |
| Cell dimensions |  |
| *a*, *b*, *c* (Å) | 173, 222, 226 |
| α, β, γ (°) | 90, 90, 90 |
| Resolution (Å) | 50.0-3.06 (3.17-3.06) ^a^ |
| *R*_sym_^b^ | 0.11 (0.196) |
| *I*/σ(*I)* | 10.1 (6.4) |
| Completeness (%) | 99.7 (99.2) |
| Redundancy | 4.4 (3.84) |
| **Refinement statistics** |  |
| Resolution (Å) | 20.0-3.06 (3.14-3.06 ) |
| No. reflections | 155016 |
| *R*_work_/*R*_free_ | 20.4/22.2 |
| No. atoms | 36918 |
| B-factors, mean B value | 47.9 |
| R.m.s deviations |  |
| Bond lengths (Å) | 0.014 |
| Bond angles (º) | 1.692 |
| Ramachandran Plot^c^ |  |
| Most favoured (%) | 96.3 |
| Allowed (%) | 2.8 |
| Disallowed (%) | 0.9 |

^a^Highest resolution shell is shown in parenthesis.

^b^R_sym_ =Σ∣(I − <I>)∣/Σ(I), where I is the observed intensity.

^c^Values calculated in CCP4 suite using Procheck.
